# High Color-Purity Green, Orange, and Red Light-Emitting Didoes Based on Chemically Functionalized Graphene Quantum Dots

**DOI:** 10.1038/srep24205

**Published:** 2016-04-06

**Authors:** Woosung Kwon, Young-Hoon Kim, Ji-Hee Kim, Taehyung Lee, Sungan Do, Yoonsang Park, Mun Seok Jeong, Tae-Woo Lee, Shi-Woo Rhee

**Affiliations:** 1 Department of Chemical and Biological Engineering, Sookmyung Women’s University, 100 Cheongpa-ro 47-gil, Yongsan-gu, Seoul 04310, Republic of Korea; 2 Department of Materials Science & Engineering, Pohang University of Science & Technology (POSTECH), 77 Cheongam-ro, Nam-gu, Pohang 37673, Republic of Korea; 3 Center for Integrated Nanostructure Physics, Institute for Basic Science (IBS), Sungkyunkwan University, 2066 Seobu-ro, Jangan-gu, Suwon 16419, Republic of Korea; 4 Department of Chemical Engineering, Pohang University of Science & Technology (POSTECH), 77 Cheongam-ro, Nam-gu, Pohang 37673, Republic of Korea; 5 Department of Energy Science, Sungkyunkwan University, 2066 Seobu-ro, Jangan-gu, Suwon 440-746, Republic of Korea

## Abstract

Chemically derived graphene quantum dots (GQDs) to date have showed very broad emission linewidth due to many kinds of chemical bondings with different energy levels, which significantly degrades the color purity and color tunability. Here, we show that use of aniline derivatives to chemically functionalize GQDs generates new extrinsic energy levels that lead to photoluminescence of very narrow linewidths. We use transient absorption and time-resolved photoluminescence spectroscopies to study the electronic structures and related electronic transitions of our GQDs, which reveals that their underlying carrier dynamics is strongly related to the chemical properties of aniline derivatives. Using these functionalized GQDs as lumophores, we fabricate light-emitting didoes (LEDs) that exhibit green, orange, and red electroluminescence that has high color purity. The maximum current efficiency of 3.47 cd A^−1^ and external quantum efficiency of 1.28% are recorded with our LEDs; these are the highest values ever reported for LEDs based on carbon-nanoparticle phosphors. This functionalization of GQDs with aniline derivatives represents a new method to fabricate LEDs that produce natural color.

Graphene quantum dots (GQDs) are graphene derivatives of nanometer size^1^,^2^; they form platelets that have an energy gap that is caused by either quantum confinement^3^,^4^ or edge effects^5^,^6^,^7^. These energy gaps give rise to a variety of optical properties, including ultraviolet-to-visible photoluminescence^8^,^9^,^10^, luminescence upconversion^11^,^12^,^13^, and hot-carrier generation^14^,^15^,^16^. GQDs can be prepared in many ways^17^, but the most common approach is use of chemical reduction of graphene oxide derived using the Hummers method or its variations^18^,^19^,^20^. Because almost all reduction processes have limited reducing power, GQDs are likely to preserve “extrinsic” chemical groups, especially oxides and nitrides. Such chemical groups could have many kinds of bonding states with different energy levels, mostly related to nonbonding (*n*) and π orbitals^21^. These energy levels interact with each other to form a variety of energy gaps, some of which generate photoluminescence that has broad linewidth.

GQDs have been used as lumophores in light-emitting diodes (LEDs)^22^. Son *et al.* demonstrated white LEDs with external quantum efficiency (EQE) = 0.18% by using zinc oxide-graphene quasi-quantum dots^23^. GQD-LEDs with alkylamine-terminated GQDs prepared by amidative cutting of graphite generated white light with EQE = ~0.1%^24^. Use of intercalated graphite has yielded deep-blue GQD-LEDs with luminous efficiency of 0.65 cd A^−1^ (ref. 25). A new approach to transfer water-soluble GQDs into a film realized blue LEDs with low turn-on voltage of 2.5 V (ref. 26). GQDs have also been used as phosphors to convert monochromatic light (usually blue) to white light^27^. These results demonstrate the possibility of using GQDs in light-emitting devices, but a main challenge for practical applications is to improve color purity and furthermore to realize green and red light.

Color purity is measured as the linewidth of light emission, often specified by full width at half maximum (FWHM). Current GQD-LEDs have very large FWHM > 100 nm, which is responsible for their inferior color purity compared to inorganic quantum dot LEDs. The main reason for such large FWHM is that GQDs have a variety of electronic states and energy gaps due to the presence of residual oxygen or nitrogen (or both) chemical groups. Here, we report unprecedented, multicolor light emission from GQDs functionalized with aniline derivatives. The aniline derivatives have no visible photoluminescence in themselves; however, they form very uniform, proper energy gaps through conjugating with GQDs to show green, orange, and red photoluminescence that has narrow linewidth. We find that the energy gap was related to the chemical properties of aniline derivatives, and its related electronic transitions were thoroughly studied by means of transient absorption and time-resolved photoluminescence spectroscopies. We finally demonstrate LEDs that use GQDs functionalized with a series of aniline derivatives to produce green, orange, and red light that has excellent color purity.

## Results and Discussion

### Synthesis and chemical analysis of GQDs

**Figure 1:**
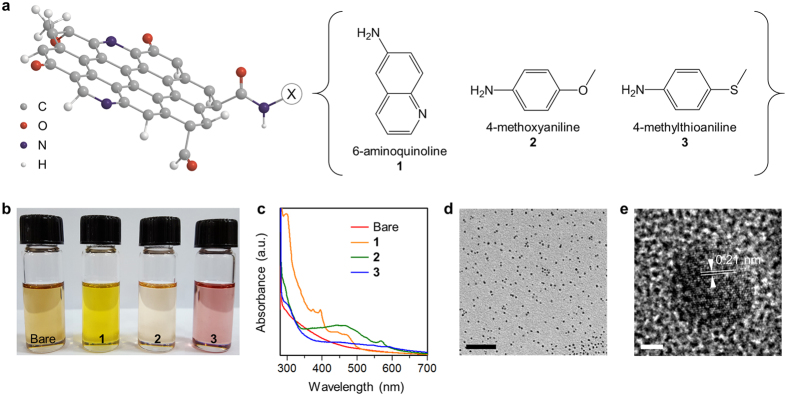
Synthesis and structural analyses. (**a**) Schematic illustration of the sturcture of GQDs and the chemical structures of aniline derivatives. (**b**) Photograph of solutions of GQDs under daylight. (**c**) Light absorption spectra of GQDs. (**d,e**) Representative TEM images of GQDs show size distribution (scale bar, 100 nm) (**d**) and graphitic structure (scale bar, 2 nm) (**e**).

Bare GQDs were prepared by amidative cutting of graphite oxide as described previously with slight modification^24^, then chemically functionalized using aniline derivatives: 6-aminoquinoline (**1**), 4-methoxyaniline (**2**), and 4-(methylthio)aniline (**3)** (Fig. 1a; details in Methods). The x-ray photoelectron spectroscopic data (Supplementary Fig. S1) show that the nitrogen content was increased by 5–10% after functionalization because these aniline derivatives all include an amine group. The sign of carbon-nitrogen (286 eV) bonding was likewise strengthened after functionalization, but the signs of carbon-carbon (284 eV) and carbon-oxygen (288.5 eV) bonding were preserved; this change indicates that the chemical structure of the graphitic core was not changed significantly (Supplementary Fig. S2). We used carbon-13 nuclear magnetic resonance (^13^C NMR) data to confirm the chemical functionalization with a series of aniline derivatives. Bare GQDs showed chemical shifts of 14.1, 22.7, 31.9, 32.6 (alkyl) and 130.4 ppm (alkene) for the oleyl group and 38.9, 39.1 and 42.8 ppm for amido carbon (Supplementary Fig. S3); these shifts indicate that oleylamine was bonded to carboxylic acid on the edge of GQDs. After functionalization, the spectra of **1**, **2** and **3** all had new lines (Supplementary Fig. S4). **1** showed two new lines, one at 142.5–150.9 ppm and the other at 127.4–137.1 ppm for the pyridine group, which are consistent with ^13^C NMR lines of 6-aminoquinoline ligand. **2** had four chemical shifts of 55.8 (methoxy), 115.1–115.8, 140.7 and 153.3 ppm (aromatic carbon) which were discovered in the ^13^C NMR of 4-methoxyaniline ligand. **3** had also four new lines at around 14.8 (methylthio), 127.8, 132.2 and 138.1–145.2 ppm (aromatic carbon), which are associated with 4-(methylthio)aniline ligand. However, **1**, **2** and **3** all retained the oleyl and amido shifts; this observation indicates that the aniline derivatives bonded to vacancies rather than replace oleylamine. This replacement would be difficult because it requires breaking amide bonds, which is difficult in our experimental conditions. Infrared spectroscopy (Supplementary Fig. S5) showed that **1**, **2** and **3** had C = O stretching (1500–1400 cm^−1^) and C–N stretching (1350–1250 cm^-1^) bands, and preserved a trace of the C–H stretching (3000–2900 cm^−1^) and N–H stretching (3500-3000 cm^−1^) bands, which all could be attributed to oleylamine on the edge of GQDs.

### Structural analysis of GQDs

After functionalization, the sample colors (Fig. 1b) were significantly changed due to the development of new light absorption bands in the range of 400–600 nm (Fig. 1c). Transmission electron microscopy (TEM) images show that the size of our GQDs is ~3 nm, and because of the presence of ligand molecules, the GQDs did not agglomerate (Fig. 1d; Supplementary Fig. S6). High-resolution TEM also visualized several signs of graphitic carbon (Fig. 1e), including the lattice spacing of 0.21 nm that corresponds to the (100) facet, and zig-zag/armchair structures found on the particle edge. However, the diffraction pattern (Supplementary Fig. S6) indicates that our GQDs have a short-range ordered structure that is very different from those of graphene or graphite, presumably due to the degradation of crystallinity by oxygen and nitrogen chemical groups. This hypothesis is further supported by Raman spectroscopy that shows no clear sign of G and D bands (Supplementary Fig. S7).

### Photoluminescence excitation and emission spectroscopy

**Figure 2:**
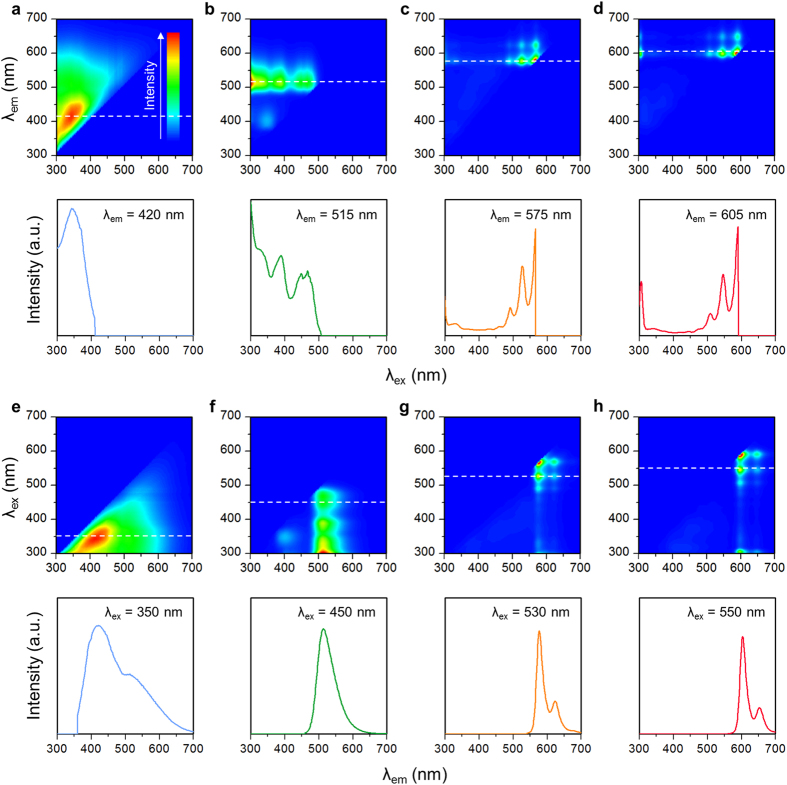
Photoluminescence spectroscopy. (**a**–**d**) Photoluminescence excitation contour maps and corresponding cross-sectional spectra at specific emission wavelengths *λ*_em_ of bare GQDs (**a**), **1** (**b**), **2** (**c**), and **3** (**d**). (**e**–**h**) Photoluminescence emission contour maps and corresponding cross-sectional spectra at specific excitation wavelengths *λ*_ex_ of bare GQDs (**e**), **1** (**f**), **2** (**g**), and **3** (**h**). Dotted lines indicate wavelengths at which cross-sectional spectra are obtained.

To investigate the effect of functionalization on the energy gap of our GQDs, we conducted photoluminescence excitation and emission spectroscopy measurements (Fig. 2). The excitation spectrum of bare GQDs consists of a single peak at wavelength *λ* = 350 nm, which corresponds to photon energy of 3.54 eV (Fig. 2a). This electronic transition may be associated with *n* orbitals of oxygen and nitrogen atoms in GQDs^21^,^22^,^24^,^28^, i.e., their *n* electrons were pumped into π* orbitals of graphitic units of GQDs by excitation light. The broad linewidth may be a result of the variety of oxygen and nitrogen chemical structures on the GQDs. Because the energy level of *n* orbitals is substantially influenced by bonding states and surroundings, the bare GQDs had many different energy gaps, each of which interacted with a specific range of excitation light; as a result, the emission linewidth was broad because it is the sum of many lines.

Functionalization of GQDs with aniline derivatives resulted in excitation bands in the visible region up to *λ* ~ 600 nm (Fig. 2b–d). These bands were composed of multiple, discrete peaks; this pattern resembles the vibrational structure of electronic transitions in dye molecules. This result suggests that the electronic structures of functionalized GQDs are largely influenced by molecule-like, discrete energy levels of the aniline derivatives. **1** had three major peaks (*λ* = 300, 380 and 460 nm) (Fig. 2b); their broad linewidths can be attributed to the large number of vibrational modes in the quinoline unit^29^. In connection with their light absorption spectra (Fig. 1c), the peak at *λ* = 300 nm can be assigned to the π → π* transition, and the peak at *λ* = 460 nm can be assigned to the *n* → π* transition. **2** had a sharp peak at *λ* ~300 nm that represents the π → π* transition, and a structured band at 450 ≤ *λ* ≤ 570 nm that consists of peaks spaced at intervals of ~40 nm (Fig. 2c), and represents the *n* → π* transition. These two transitions are separated by a forbidden gap; i.e., the functionalized GQDs have the discrete energy levels. **3** also exhibits a π → π* peak at *λ* = 305 nm and *n* → π* band at 470 ≤ *λ* ≤ 590 nm (Fig. 2d). Its *n* → π* band position is red-shifted by 20 nm with respect to **2**, presumably because sulfur atoms near positive carbon in **3** can act as a stronger π donor than oxygen atoms in **2** and may possibly result in a bathochromic shift^30^.

The emission spectrum of bare GQDs shows a long-tailed, asymmetric peak at *λ* = 420 nm (Fig. 2e). This typical peak shape has been considered to indicate that GQDs possess various photoluminescence centers that have distinct chemical structures and correspondingly different singlet ground (S_0_)–first-excited state (S_1_) gaps^31^,^32^. After functionalization, **1** exhibits a relatively narrow, single peak (FWHM ~50 nm) at *λ* = 510 nm (Fig. 2f). The peak position remained constant regardless of excitation light; i.e., Kasha’s rule was met. The emission spectrum of **2** shows a very narrow peak (FWHM ~20 nm) at *λ* = 570 nm with a minor peak at *λ* = 620 nm (Fig. 2g). This double-peaked photoluminescence has been often observed in polyaromatic systems^33^,^34^, and in our case, the energy difference (~0.1 eV) may correspond to the C–C inter-ring or intra-ring stretch modes. Similarly, **3** had a major peak at *λ* = 605 nm and a minor peak at *λ* = 655 nm (Fig. 2h). The similarity between the emission spectra of **2** and **3** may be due to the similarity in their ligands. The photograph of the photoluminescence of our GQDs is shown in Supplementary Fig. S8.

### Effect of the degree of functionalization

Our photoluminescence spectroscopic data have showed that functionalization of GQDs with aniline derivatives could cause a dramatic change in their electronic structures. To understand the role of aniline derivatives in photoluminescence, we controlled the degree of functionalization by varying the reaction time (Supplementary Figs S9 and 10). Here, the degree of functionalization was determined by means of x-ray photoelectron spectroscopy (Supplementary Figs S11 and 12). As the reaction time was increased, the intensity of “intrinsic” emission of bare GQDs at 350 ≤ *λ* ≤ 450 nm was decreased while that of “extrinsic” emission of functionalized GQDs at *λ* > 500 nm was increased. This result strongly implies that intrinsic photoluminescence centers were converted to extrinsic photoluminescence centers through our functionalization. Importantly, the energy gap of photoluminescence centers was independent of the degree of functionalization; i.e., the photoluminescence peak wavelengths were determined entirely by the chemical structure of the aniline derivatives, which indicates that the extrinsic photoluminescence was due to the formation of extrinsic energy level, and not to an auxochromic effect or to any other indirect effects.

### Transient absorption and time-resolved photoluminescence spectroscopy

**Figure 3:**
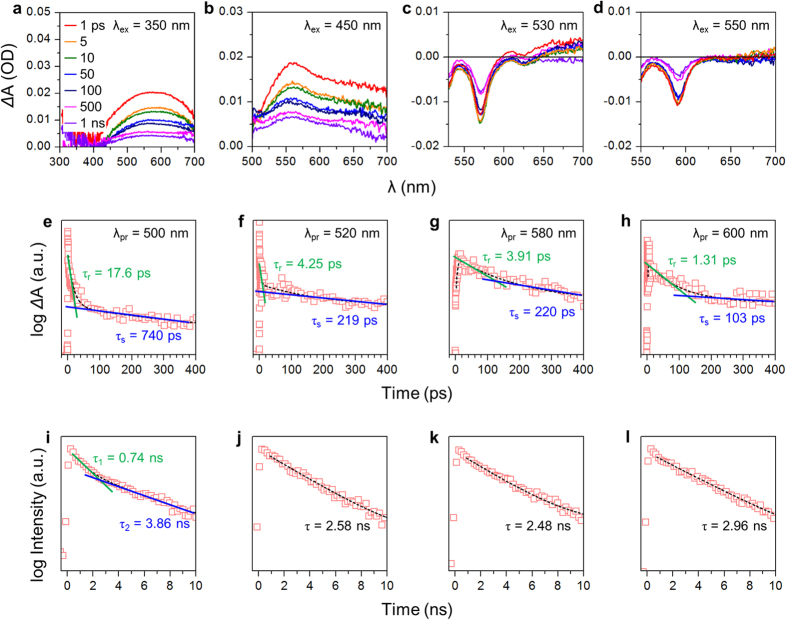
Transient absorption and time-resolved photoluminescence spectroscopy. (**a**–**d**) Transient absorption spectra at specific excitation wavelengths λ_ex_ and (**e**–**h**) corresponding time-correlated excited-state electron decay profiles at specific probe wavelengths λ_pr_ of bare GQDs (**a**,**e**), **1** (**b**,**f**), **2** (**c**,**g**), and **3** (**d**,**h**). (**i**–**l**) Time-resolved photoluminescence spectra at specific excitation wavelengths (λ_ex_) of bare GQDs (**e**), **1** (**f**), **2** (**g**), and **3** (**h**).

To further explore the electronic transition in our GQDs, we performed transient absorption spectroscopy measurements. Bare GQDs exhibited excited-state absorption (ΔA > 0) on all time scales (Fig. 3a); this observation implies the existence of numerous accessible states that are energetically near the first-excited state^31^,^35^. Similarly, **1** had ΔA > 0 on all time scales, but with a peak at around *λ* = 550 nm (Fig. 3b); the shift might indicate that **1** is more molecule-like than bare GQDs, and its quinoline unit of many vibrational modes caused very strong excited-state absorption. **2** exhibits ground-state bleaching signals (ΔA < 0) with a sharp peak at *λ* = 570 nm (Fig. 3c), which corresponds to its major excitation peak wavelength (Fig. 2c). Such localized ground-state bleaching has been commonly observed in dye molecules^36^,^37^, and proves the existence of an energy state that is accessible to electrons that had been photoexcited at *λ* = 570 nm. This kind of ground-state bleaching was also observed for **3** at *λ* = 590 nm (Fig. 3d).

We traced the change of ΔA in the time domain at a specific probe wavelength (Fig. 3e–h). The decay curves were fitted using bi-exponential functions (Supplementary Information, Table S3); the applicability of this model indicates that all of the functionalized GQDs undergo two distinguishable transient behaviors: an early rapid (few picoseconds) decay (τ_r_) for intra-band relaxation through carrier-phonon scattering, and a subsequent slow (hundreds of picoseconds) decay (τ_s_) for carrier trapping to photoluminescence centers (or ligands). The carrier trapping time for bare GQDs (740 ps, Fig. 3e) was 4–7 times longer than those of with **1** (219 ps, Fig. 3f), 2 (220 ps, Fig. 3g), and 3 (103 ps, Fig. 3h). The relatively long trapping time in bare GQDs can be attributed to the presence on them of many kinds of chemical groups that can provide trapping states independently. The extended trapping time was also observed in the time-resolved photoluminescence (TRPL) spectrum of bare GQDs (Fig. 3i), which was fitted to bi-exponential functions with the first time constant τ_1_ = 0.74 ns identical to the carrier trapping time in the bi-exponential model of the change of ΔA over time. In contrast, the TRPL spectra of functionalized GQDs (Fig. 3j–l) were fitted to single-exponential functions with time constants τ of a few nanoseconds; the applicability of a single function indicates that recombination through specific states (e.g. S_1_ → S_0_) may occur dominantly. We finally illustrated the electronic structures of our GQDs and viable electronic transitions that could take place (Supplementary Fig. S13).

### LED demonstration

**Figure 4:**
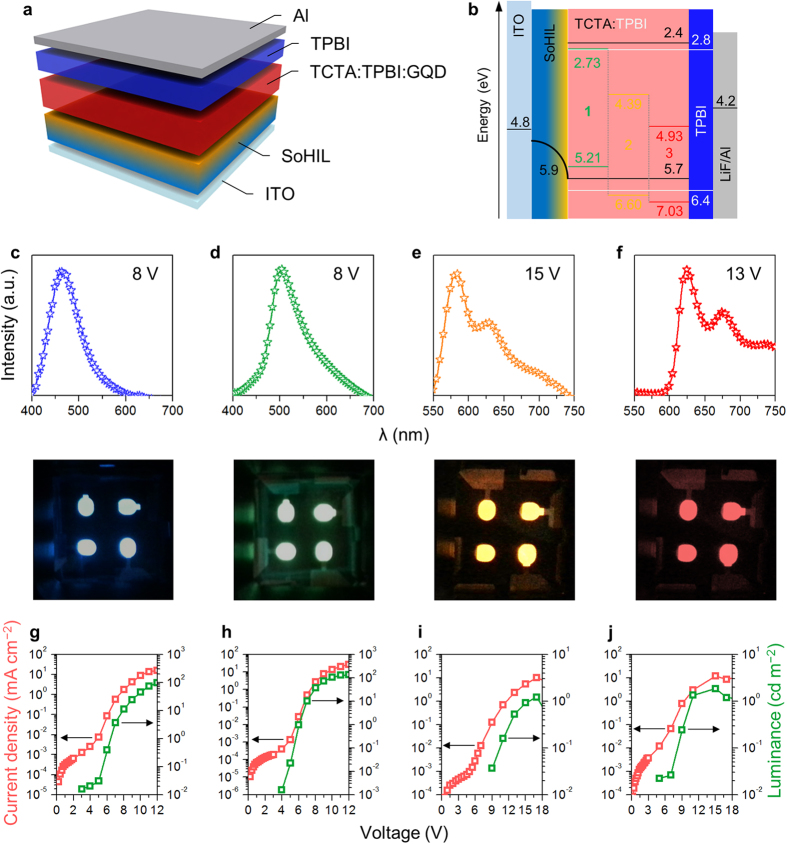
LED demonstration. (**a**) Structure of LEDs. (**b**) Energy levels in LEDs. (**c**–**f**) Electroluminescence spectra (top) and photographs (bottom) of host-only (**c**), **1**- (**d**), **2**- (**e**), and **3**-LEDs (**f**). Current density and luminance of host-only (**g**), **1**- (**h**), **2**- (**i**), and **3**-LEDs (**j**).

We demonstrated LEDs that use 1,3,5-tris(N-phenylbenzimidizol-2-yl)benzene (TPBI):tris(4-carbazoyl-9-ylphenyl)amine (TCTA) in 1:1 weight ratio as a co-host, and our functionalized GQDs as a dopant in an emitting layer (EML) (Fig. 4a). The concentrations of **1**, **2**, and **3** in the host matrix were 10, 20, and 20 wt%, respectively. Each device also included a self-organized polymeric hole injection layer (SOHIL), which is composed of a conventional poly(3,4-ethylenedioxythiphene):poly(styrene sulfonate) (PEDOT:PSS) and perfluorinated polymeric acid, tetra-fluoroethylene-perfluoro-3,6-dioxa-4-methyl-7-octene-sulfonic acid copolymer (PFI)^38^,^39^,^40^,^41^. Due to self-organization of PFI during spin coating, the SOHIL has a work function that increases gradually from 5.2 eV at the bottom to 5.95 eV at the top; this gradient induces efficient hole injection into EML (TCTA:TPBI:GQD) by reducing the hole-injection barrier. The large proportion of PFI at the top of the SOHIL also prevents exciton quenching at the interface between PEDOT:PSS and EML, and consequently increases the luminescence efficiency of devices^40^,^41^. Under a certain electrical bias, electrons are pumped through Al/LiF/TPBI cathodes and holes are injected through ITO/SOHIL anodes (Fig. 4b). These injected carriers are then transferred into a co-host system in the EML. The co-host system that incorporates hole-transporting TCTA and electron-transporting TPBI has facilitated direct carrier injection by broadening the recombination zone^42^, which can increase the device efficiency, and generate a pure electroluminescence spectrum from GQD dopants. For functionalized GQDs, the highest occupied molecular orbital (HOMO) levels were determined by means of ultraviolet photoelectron spectroscopy (Supplementary Fig. S14) and the lowest unoccupied molecular orbital (LUMO) levels were deduced from Kelvin probe analysis results (Supplementary Fig. S15) and photoluminescence excitation onset wavelengths (details in Supplementary Information).

The emission spectra of the LEDs were affected by the functionalization. The host-only LEDs emitted deep blue light (peak at *λ* = 460 nm, FWHM = 60 nm; Fig. 4c); *L*_max_ = ~100 cd m^−2^ and EQE = ~0.4% were achieved at 10 V (Fig. 4g; Supplementary Fig. S16). The Commission Internationale de l’Éclairage (CIE) coordinates were (0.192, 0.212; Supplementary Fig. S17). The LEDs employing **1** emitted green light (peak at *λ* = 510 nm, FWHM = 80 nm; Fig. 4d) with *L*_max_ = 390 cd m^−2^ and EQE = 1.28% (Fig. 4h; Supplementary Fig. S18). The CIE coordinates were (0.286, 0.496; Supplementary Fig. S19). The electroluminescence spectra of **1**-LEDs overlapped the photoluminescence spectrum and was not affected by applying bias (Supplementary Fig. S18); these traits indicate that the TCTA:TPBI co-host efficiently transfers carriers into **1**. To our best knowledge, this is the highest efficiency ever reported for LEDs based on carbon nanoparticles as a phosphor (Supplementary Information, Table S5). The electroluminescence spectrum of the **2-**LEDs shows two peaks: a major one at *λ* = 590 nm and a lesser one at *λ* = 630 nm, where FWHM of the major peak is ~50 nm (Fig. 4e). This device showed *L*_max_ = ~3 cd m^−2^ and EQE = ~0.1% (Fig. 4i; Supplementary Fig. S20). The CIE coordinates were (0.567, 0.432), which is located at around the color boundary between orange and amber (Supplementary Fig. S21). The **3-**LEDs emitted red light (peak at *λ* = 620 nm, FWHM ~50 nm; Fig. 4f) with *L*_max_ = ~2 cd m^−2^ and EQE = ~0.1% (Fig. 4j; Supplementary Fig. S22). The CIE coordinates were (0.682, 0.318), which indicate that the emission color was very saturated and pure (Supplementary Fig. S23). These colors emitted by these three devices are very close to the National Television System Committee standard colors. The poor efficiency of devices with **2** and **3** may be due to several factors such as the presence of methoxy and methylthio groups that block electron injection due to their high polarity^43^,^44^,^45^, the lower fluorescence quantum yields (**2** = 22%; **3** = 21%) than **1** (29%; Supplementary Fig. S24), and the deeper HOMO levels (**2** = 6.60 eV; **3** = 7.03 eV) than **1** (5.21 eV). These results imply that direct charge injection from host to guest is an important mechanism of electroluminescence of LEDs based on GQDs^24^.

## Conclusions

Recent trials to demonstrate GQD-based LEDs have achieved a considerable success, but a remaining major challenge is to achieve high color purity (i.e., narrow linewidth of emitted light). To overcome these challenges, we chemically functionalized GQDs with a series of aniline derivatives. After this functionalization, our GQDs showed dramatic narrowing of photoluminescence linewidths. This improvement could be attributed to the formation of new extrinsic energy levels as a result of interaction between intrinsic energy levels of GQDs and aniline derivatives. Due to these extrinsic energy levels, the LEDs that use our functionalized GQDs as lumophores exhibited green, orange, and red electroluminescence that has narrow linewidths (FWHM < 80 nm) and high color purity. The best *L*_max_ = 390 cd m^−2^ and EQE = 1.28% (current efficiency = 3.47 cd A^−1^) were recorded with our green LED. Our devices are still inferior to the state-of-the-art LEDs based on inorganic quantum dots; however, considering resource depletion and environmental pollution related to use of rare-earth and heavy metals, these functionalized GQDs may have strong potential as clean light sources in future displays.

## Methods

### Chemicals

All chemicals were purchased from Aldrich and used as received unless otherwise specified.

### Synthesis of bare GQDs

A single-neck round-bottom flask was charged with ~20 μm graphite powder (100 mg) and concentrated nitric acid (40 ml). This mixture was heated at 100 °C for 12 h. The resulting transparent pale-green solution was added to excess water (60 ml) and filtered by suction to remove foam. The filtered solution was then transferred into a three-neck round-bottom flaks that had been charged with oleylamine (1 ml) and 1-octadecene (9 ml). The mixture was heated at 250 °C for 3 h under vigorous stirring. After cooling down to 100 °C, to the solution was added hydrazine hydrate (2 mL), and stirred for 3 h. The resulting dark-brown solution was precipitated with excess methanol (30 ml), isolated by centrifugation (3,000 rpm for 15 min), and re-dispersed in hexane (3 ml). This process was repeated three times; the purified solution was concentrated by a rotary evaporator and kept in a vacuum oven at 80 °C until it was used.

### Chemical functionalization

Bare GQDs (10 mg) were dissolved in toluene (10 mL). To the solution was added 6-aminoquinoline (1.44 g, 10 mmol), 4-methoxyaniline (1.23 g, 10 mmol), or 4-(methylthio)aniline (1.39 g, 10 mmol) to give **1**, **2**, or **3**, respectively. The mixture was heated at 120 °C for 12 h under vigorous stirring. The resulting solid was dissolved in toluene (5 ml) and dialyzed against excess toluene by using Spectra/Por Biotech Cellulose Ester dialysis tubes (100–500 Da). The dialyzed solution was concentrated by a rotary evaporator and kept in a vacuum oven at 80 °C until it was used.

### Chemical and structural analyses

X-ray photoelectron spectroscopy was performed by using an Escalab 250 spectrometer with an Al x-ray source (1486.6 eV). Nuclear magnetic resonance spectra were recorded on a Bruker DRX500 spectrometer (500 MHz). Infrared spectroscopy was performed by using a Nicolet 6700 FT-IR spectrometer equipped with a demountable cell (Part Num. 162–3600) with a pair of KBr windows (Pike Technologies). TEM was performed using a Jeol JEM-2200FS equipped with a Cs corrector. Raman spectroscopy was performed by using a Witec Alpha 300 R spectrometer with a laser excitation wavelength of 785 nm.

### Optical characterization

Light absorption spectra were recorded on a Scinco S-3100 spectrophotometer. Photoluminescence excitation and emission spectra were recorded on a Jasco FP-8500 fluorometer.

### Transient absorption and time-resolved photoluminescence spectroscopy

The photo-generated carrier dynamics was measured by a pump-probe system (Helios, Ultrafast system). The 1-kHz Ti:sapphire amplifier pumped a 5-mm sapphire window to generate a white-light-continuum in the visible range for the probe beam, and an optical parametric amplifier (OPA, TOPAS prime, Coherent) which pumped by the Ti:sapphire amplifier was used as the pump beam. We used different pump wavelengths for the resonant excitation of *n* → π* band; 350 nm for bare GQDs, 450 nm for **1**, 525 nm for **2**, and 540 nm for **3**. To measure the time-resolved photoluminescence (TRPL), a Hamamatsu c11200/PLP streak camera with 10-ps time resolution was used. An Acton 2300i spectrograph was combined with the streak camera to spectrally resolve the TRPL. The OPA beam was used as a marker of the time zero for the optical pump.

### Energy level analyses

Kelvin probe force microscopy was performed using an SKP5050 Scanning Calvin probe (KP Technology). Ultraviolet photoelectron spectroscopy was conducted in an ultra-high-vacuum chamber equipped with a VUV-5000 generator (40.8 eV He II laser) and a SES-100 detector.

### Quantum yield measurements

2 mm × 10 mm QS-grade quartz cuvettes (Jasco Parts Center 6808-H250A) were used. Absolute quantum yields were recorded on a Jasco FP-8500 fluorometer equipped with a 100-mm integrating sphere setup (ILF-835), and calculated using Jasco Spectra Manager II Software.

### Fabrication and characterization of GQD-based LEDs

ITO-patterned glass was sonicated in acetone and 2-isopropanol for 15 min respectively, and boiled in 2-isopropanol to remove the residues and dusts. Then, it was treated with UV-ozone to make the surface hydrophilic and clean. After SOHIL were spin-coated and baked for 30 min for 150 °C to give the 40-nm thickness, substrate was moved to the N_2_-filled glove box. EML solution containing ligand-exchanged GQD dopants, TCTA as a hole transporting host and TPBI as an electron transporting host in tetrahydrofuran were spin-coated to give 40-nm thickness and baked to remove the residual solvent and make a compact and uniform film. Then TPBI (50 nm), LiF (1 nm) and Al (100 nm) were deposited sequentially deposited in a high vacuum chamber (<10^−7^ Torr). The current-voltage-luminance characteristics were measured using a Keithley 236 source measurement unit and a Minolta CS2000 spectroradiometer.

## Additional Information

**How to cite this article**: Kwon, W. *et al.* High Color-Purity Green, Orange, and Red Light-Emitting Didoes Based on Chemically Functionalized Graphene Quantum Dots. *Sci. Rep.* **6**, 24205; doi: 10.1038/srep24205 (2016).

## Supplementary Material

Supplementary Information
