# Corrigendum: High Color-Purity Green, Orange, and Red Light-Emitting Diodes Based on Chemically Functionalized Graphene Quantum Dots

**DOI:** 10.1038/srep27827

**Published:** 2016-06-20

**Authors:** Woosung Kwon, Young-Hoon Kim, Ji-Hee Kim, Taehyung Lee, Sungan Do, Yoonsang Park, Mun Seok Jeong, Tae-Woo Lee, Shi-Woo Rhee

Scientific Reports 6: Article number: 2420510.1038/srep24205; published online: 04062016; updated: 06202016

The original version of this Article contained an error in the title of the paper, where the word “Diodes” was incorrectly given as “Didoes”. This error has now been corrected in the PDF and HTML versions of the Article.

