# Supplementary material for: High Color-Purity Green, Orange, and Red Light-Emitting Didoes Based on Chemically Functionalized Graphene Quantum Dots
Source: Sci Rep. 2016 Apr 6;6:24205. doi: 10.1038/srep24205 (PMC4822170; doi:10.1038/srep24205)
Supplement: Supplementary Information [file srep24205-s1.doc]

Supplementary Information for

High Color-Purity Green, Orange, and Red Light-Emitting Didoes Based on Chemically Functionalized Graphene Quantum Dots

Woosung Kwon^1§^, Young-Hoon Kim^2§^, Ji-Hee Kim^3§^, Taehyung Lee^4§^, Sungan Do^4^, Yoonsang Park^4^, Mun Seok Jeong^3,5^, Tae-Woo Lee^2★^ and Shi-Woo Rhee^1,4★^

^1^Department of Chemical and Biological Engineering, Sookmyung Women’s University, 100 Cheongpa-ro 47-gil, Yongsan-gu, Seoul, Republic of Korea 04310

^2^Department of Materials Science & Engineering, Pohang University of Science & Technology (POSTECH), 77 Cheongam-ro, Nam-gu, Pohang, Republic of Korea 37673

^3^Center for Integrated Nanostructure Physics, Institute for Basic Science (IBS), Sungkyunkwan University, 2066 Seobu-ro, Jangan-gu, Suwon, Republic of Korea 16419

^4^Department of Chemical Engineering, Pohang University of Science & Technology (POSTECH), 77 Cheongam-ro, Nam-gu, Pohang, Republic of Korea 37673

^5^Department of Energy Science, Sungkyunkwan University, 2066 Seobu-ro, Jangan-gu, Suwon, Republic of Korea 440-746

^§^These authors contributed equally to this work.

**Corresponding author footnote**

^★^Corresponding author.

Tel: +82-54-279-2151; Fax: +82-54-279-2399; E-mail: twlee@postech.ac.kr (T-.W.L.)

Tel: +82-54-279-2265; Fax: +82-54-279-8619; E-mail: srhee@postech.ac.kr (S-.W.R.)

**Contents**

Methods for sample preparation 1

Figure S1 | X-ray photoelectron spectroscopy (N1s) 3

Figure S2 | X-ray photoelectron spectroscopy (C1s) 4

Figure S3 | ^13^C NMR data of bare GQDs 5

Figure S4 | ^13^C NMR data of functionalized GQDs 6

Figure S5 | Infrared spectroscopy 7

Figure S6 | TEM images, size statistics and diffraction pattern 8

Figure S7 | Raman spectroscopy 9

Figure S8 | Photograph of the photoluminescence of GQDs 10
Figure S9 | Degree of functionalization (photoluminescence excitation spectroscopy) 11
Figure S10 | Degree of functionalization (photoluminescence emission spectroscopy) 12
Figure S11 | Degree of functionalization (x-ray photoelectron spectroscopy) 13
Figure S12 | Degree of functionalization (x-ray photoelectron spectroscopy, C1s) 14
Figure S13 | Electronic structures and transitions 16

Figure S14 | Ultraviolet photoelectron spectroscopy 17
Figure S15 | Kelvin probe analysis 18

Figure S16 | Characteristics of host-only LEDs 20

Figure S17 | CIE coordinates of host-only LEDs 21
Figure S18 | Characteristics of **1** LEDs 22

Figure S19 | CIE coordinates of **1** LEDs 23

Figure S20 | Characteristics of **2** LEDs 24

Figure S21 | CIE coordinates of **2** LEDs 25

Figure S22 | Characteristics of **3** LEDs 26

Figure S23 | CIE coordinates of **3** LEDs 27

Figure S24 | Fluorescence quantum yields 28

Table S1 | Atomic ratios of GQDs 3

Table S2 | Degree of functionalization (atomic ratios of functionalized GQDs) 15

Table S3 | Decay times of photoexcited carriers 16

Table S4 | Estimation of energy levels 18

Table S5 | Comparison with previous literatures 19

References for Supplementary Information 29

**Methods for sample preparation**

**TEM.** Vacuum-dried GQD samples (1 mg) were dissolved in hexane (1 ml). The solution (10 μl) was dropped onto a CF300-Cu TEM grid (Electron Microscopy Sciences) and dried for 12 h at 80 ºC in vacuum.

**Raman spectroscopy.** Vacuum-dried GQD samples (10 mg) were dissolved in toluene (1 ml). An aliquot of the solution (100 μl) was spin-cast on gold-coated silicon substrates and dried for 12 h in air.

**X-ray photoelectron spectroscopy.** Vacuum-dried GQD samples (10 mg) were dissolved in toluene (1 ml). An aliquot of the solution (100 μl) was spin-cast on gold-coated silicon substrates and dried for 12 h in air.

**Nuclear magnetic resonance.** Vacuum-dried GQD samples (100 mg) were dissolved in deuterated chloroform (1 ml). The solution was transferred into 600 MHz nuclear magnetic resonance sample tubes (Optima) and sealed by Teflon tapes.

**Infrared spectroscopy.** Vacuum-dried GQD samples (100 mg) were dissolved in anhydrous toluene (1 ml). An aliquot of the solution (100 μl) was dropped onto a KBr window (Pike Technologies), dried for 12 h under high-purity nitrogen flow and sandwiched with another KBr window. This pair of KBr windows with GQD samples was mounted on a demountable cell.

**Kelvin probe force microscopy.** Vacuum-dried GQD samples (10 mg) were dissolved in toluene (1 ml). An aliquot of the solution (100 μl) was spin-cast on gold-coated silicon substrates and dried for 12 h in air. The probe was calibrated by using a gold substrate.

**Ultraviolet photoelectron spectroscopy.** Vacuum-dried GQD samples (10 mg) were dissolved in toluene (1 ml). An aliquot of the solution (100 μl) was spin-cast on gold-coated silicon substrates and dried for 12 h in air.


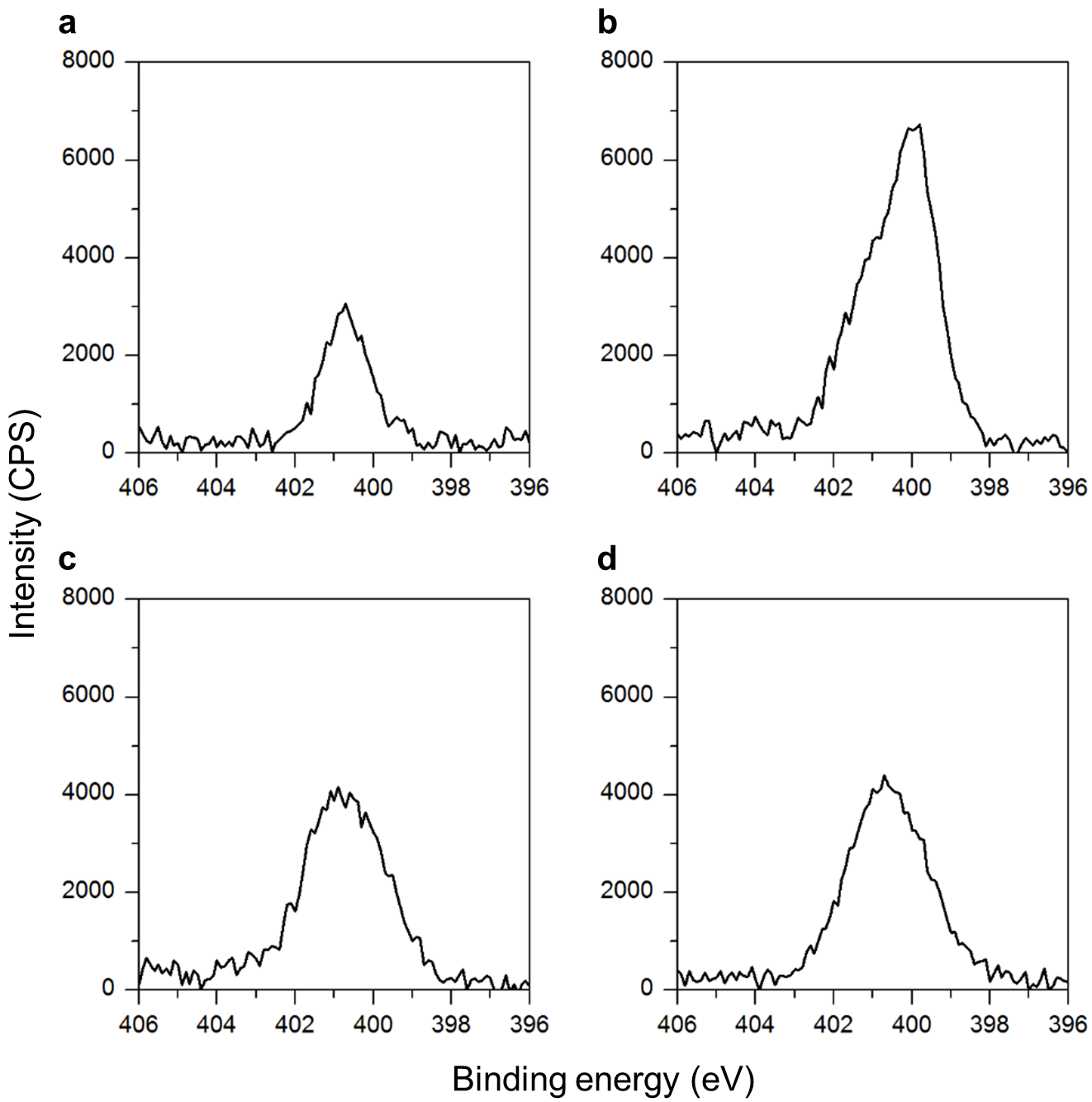


**Figure S1.** X-ray photoelectron spectra (N1s) of bare GQDs (**a**), **1** (**b**), **2** (**c**) and **3** (**d**).

**Table S1.** Atomic ratios of our GQDs.

| Sample | Atomic ratio (%) | | | |
| --- | --- | --- | --- | --- |
|  | C | N | O | S |
| Bare | 91 | 1.03 | 5.84 | ~0 |
| **1** | 73.75 | 12.47 | 11.79 | ~0 |
| **2** | 78.38 | 7.02 | 12.88 | ~0 |
| **3** | 81.59 | 6.63 | 8.16 | 1.83 |

**
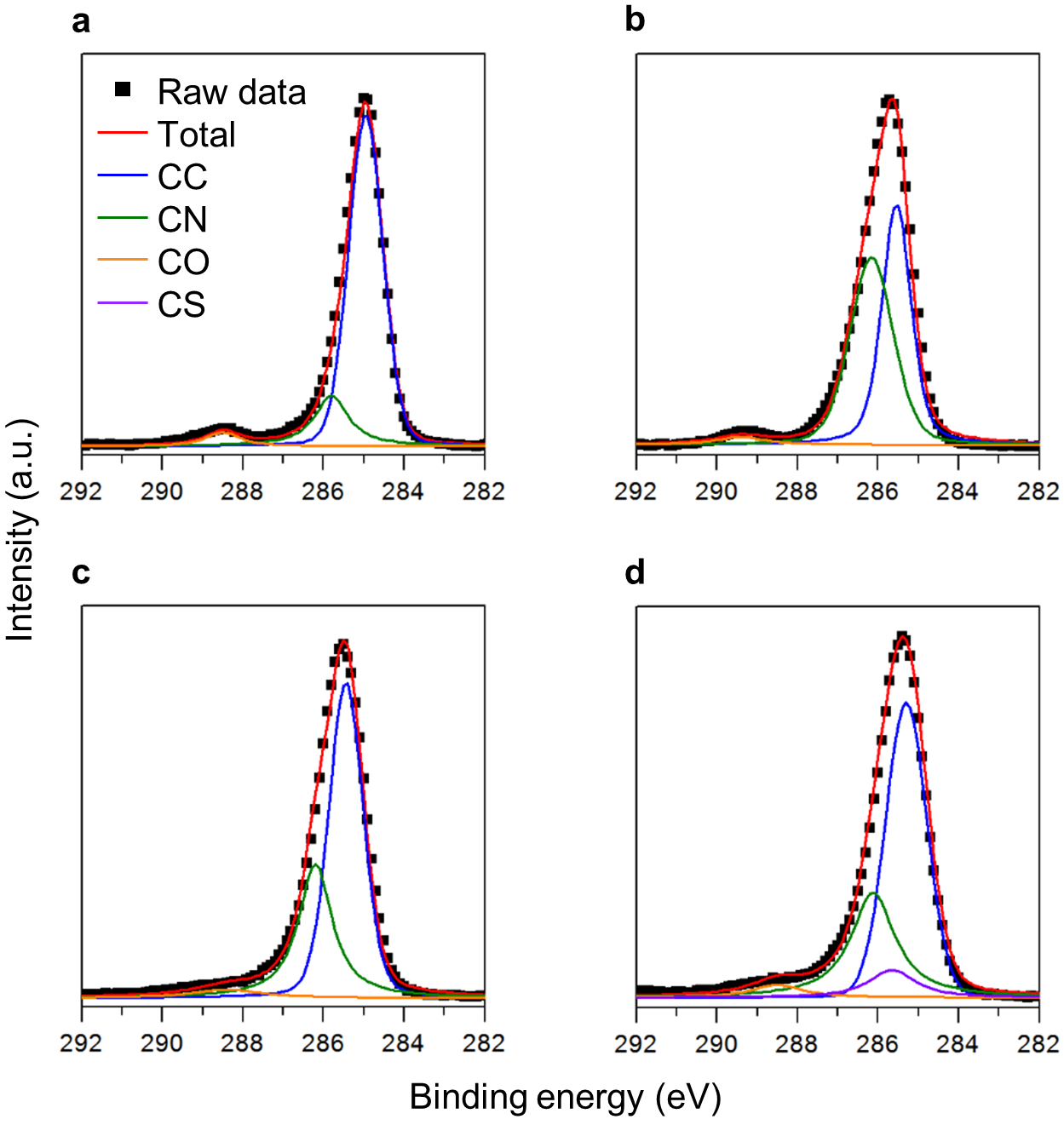
**

**Figure S2.** X-ray photoelectron spectra (C1s) of bare GQDs (**a**), **1** (**b**), **2** (**c**) and **3** (**d**).

The x-ray photoelectron spectroscopy data were deconvoluted on the basis of “Handbook of X-ray Photoelectron Spectroscopy” by Moulder et al. We detected a slight shift (~2 eV) in the binding energy of the chemical bondings with respect to the literature values (CC = 284.5 eV; CN = 286-288 eV; CO = 286-290 eV; CS = 286-287 eV) due to a high dipole moment of our GQDs. The CS bonding was only found in **3** due to its (methylthio)aniline ligands.


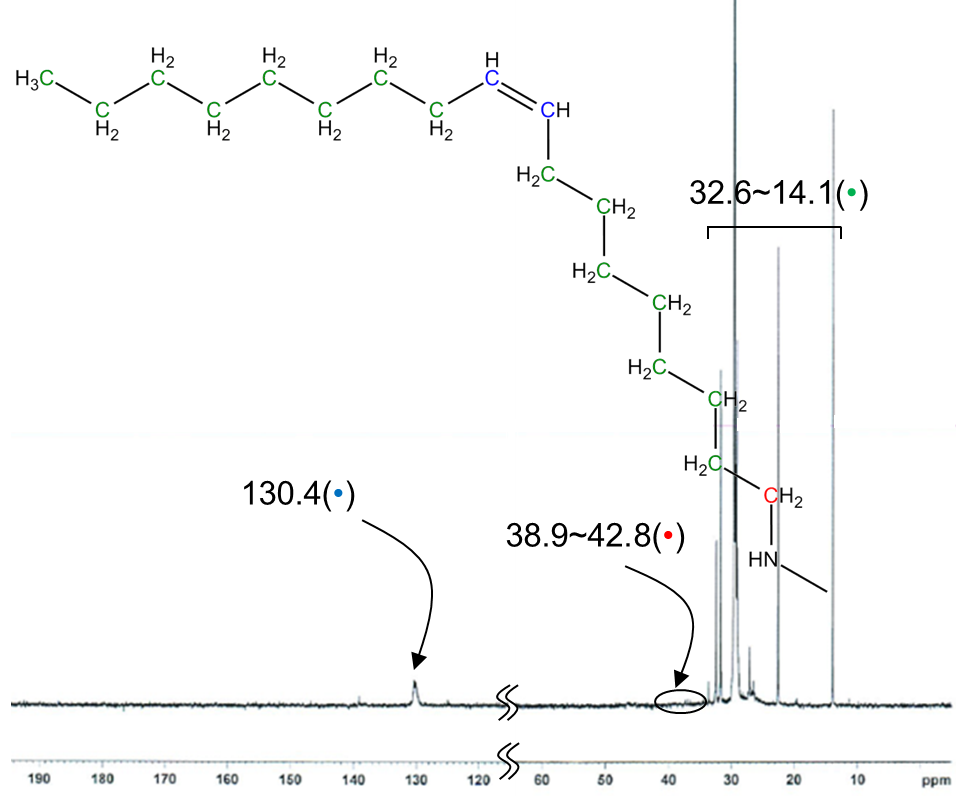


**Figure S3.** ^13^C NMR data of bare GQDs.


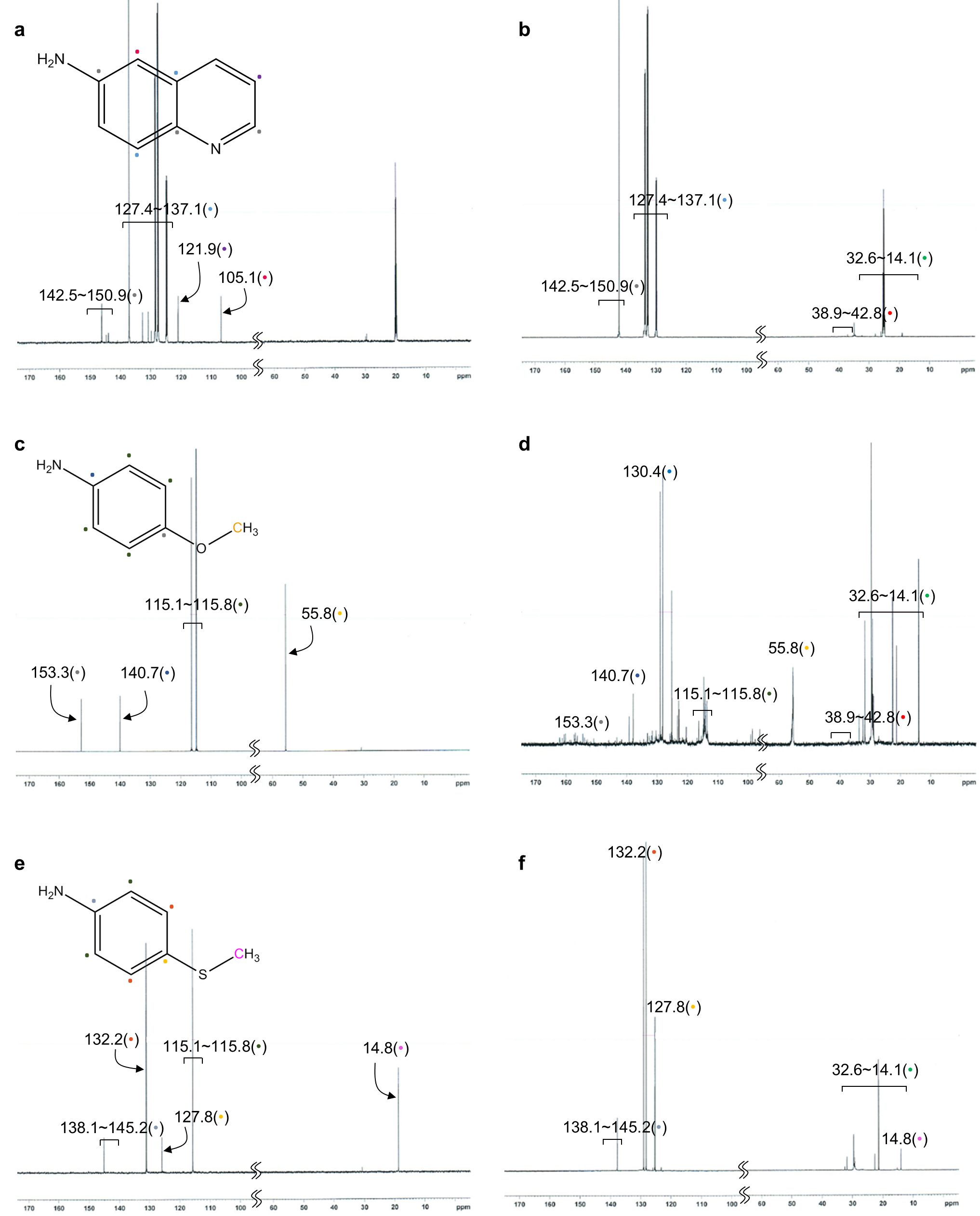


**Figure S4.** ^13^C NMR data of 6-aminoquinoline (**a**), **1** (**b**), 4-methoxyaniline (**c**), **2** (**d**), 4-(methylthio)aniline (**e**) and **3** (**f**).


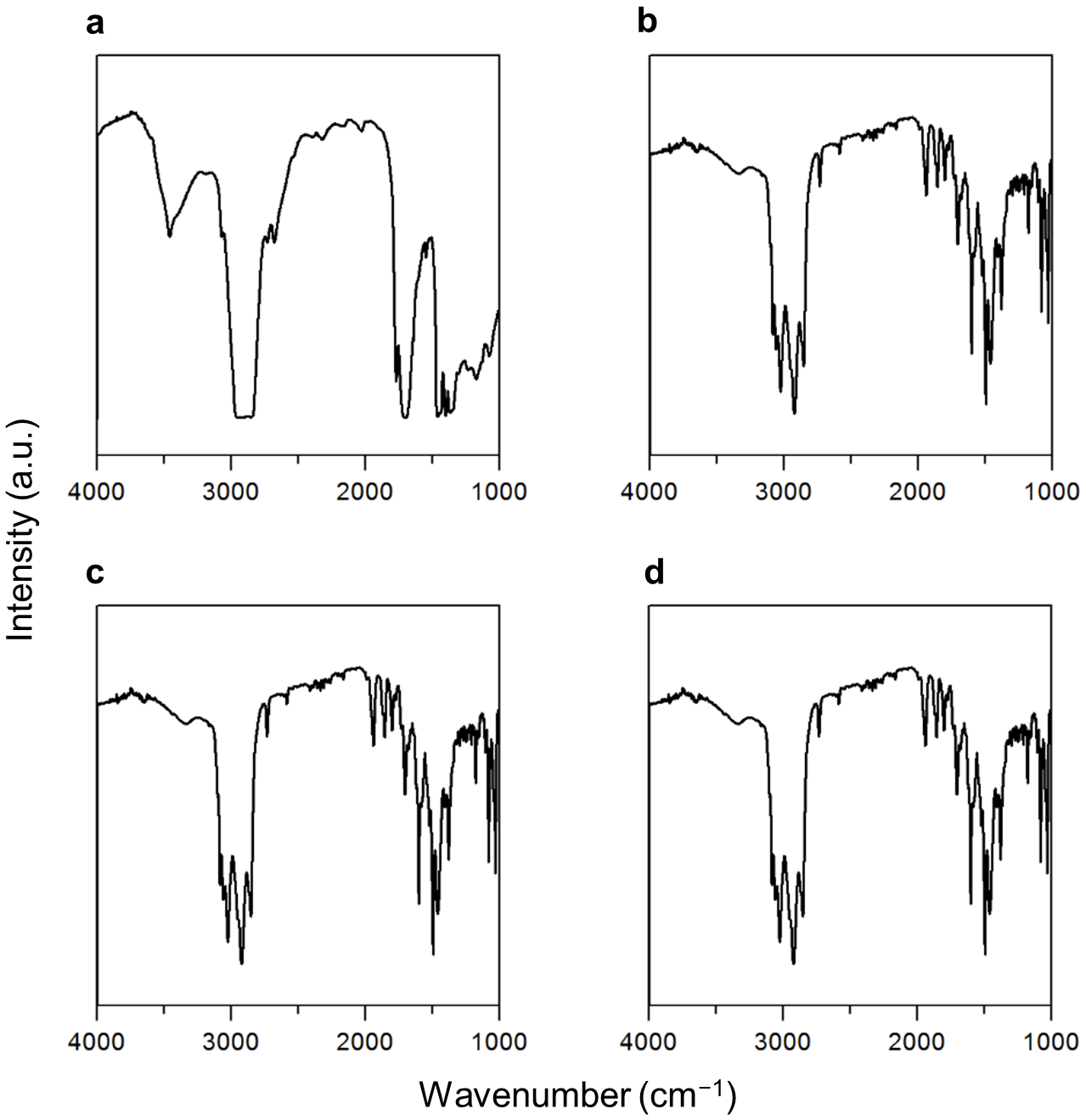


**Figure S5.** Infrared spectra of bare GQDs (**a**), **1** (**b**), **2** (**c**) and **3** (**d**).

The peak at around 3500 cm^−1^ for NH stretching is attributed to the amine group of oleylamine and diminished after the functionalization. The peak at around 3000 cm^−1^ represents CH stretching. The band of peaks at around 1500 cm^−1^ for CN stretching, NH bending of amide, C=O stretching of amide is attributed to the aniline derivatives that are bonded with the edge functional groups of our GQDs. The peak at around 1200 cm^−1^ for CO stretching is attributed to carboxyl groups such as carboxylic acid and aldehyde that exist at the edge of the GQDs.


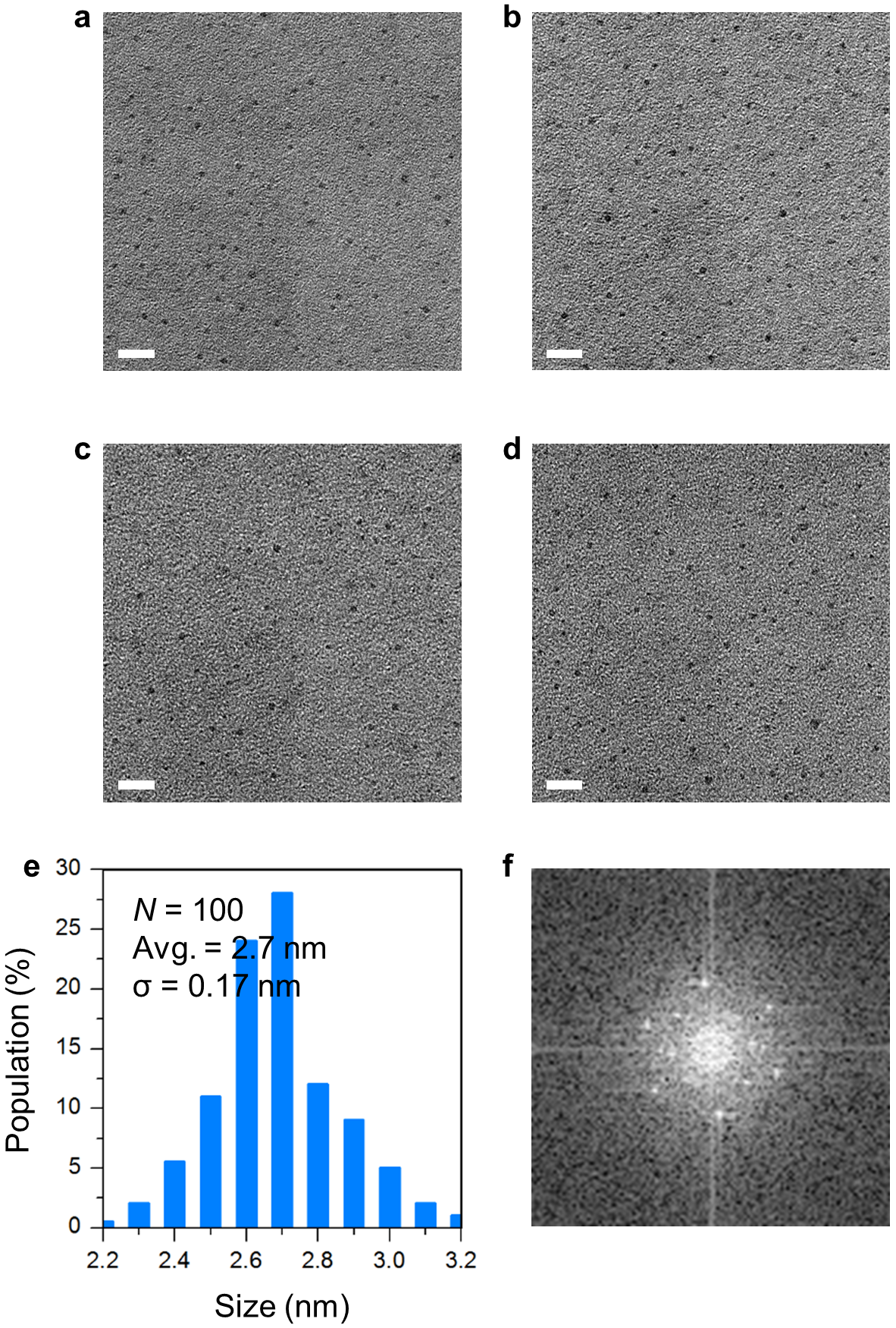


**Figure S6.** (**a**−**d**) TEM images of bare GQDs (**a**), **1** (**b**), **2** (**c**) and **3** (**d**) for size statistics (scale bars, 10 nm). (**e**) Size distribution of all samples. Population ratios below 0.5% are not shown. (**f**) Diffraction pattern of GQDs.


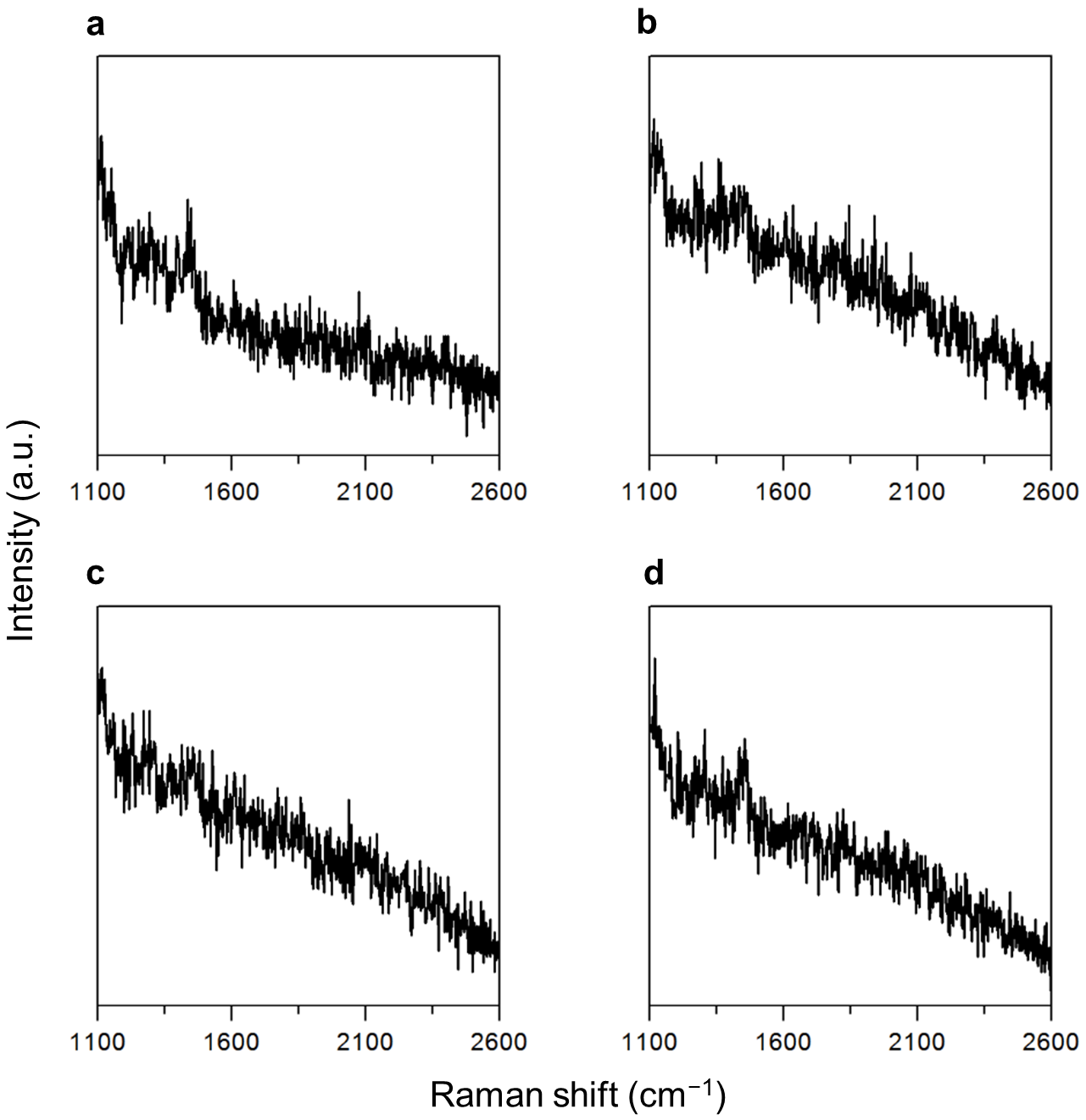


**Figure S7.** Raman spectra of bare GQDs (**a**), **1** (**b**), **2** (**c**) and **3** (**d**).


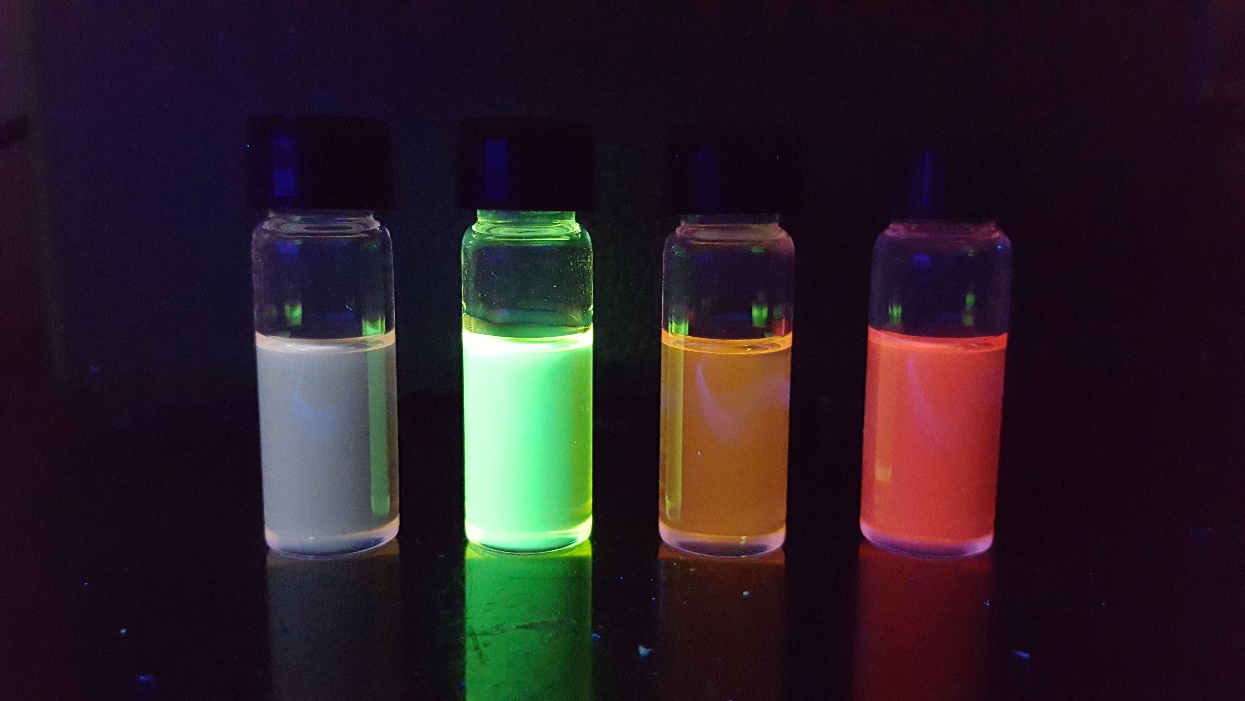


Bare

**1**

**2**

**3**

**Figure S8.** Photo of photoluminescence of our GQDs under UV light (312 nm) irradiation.


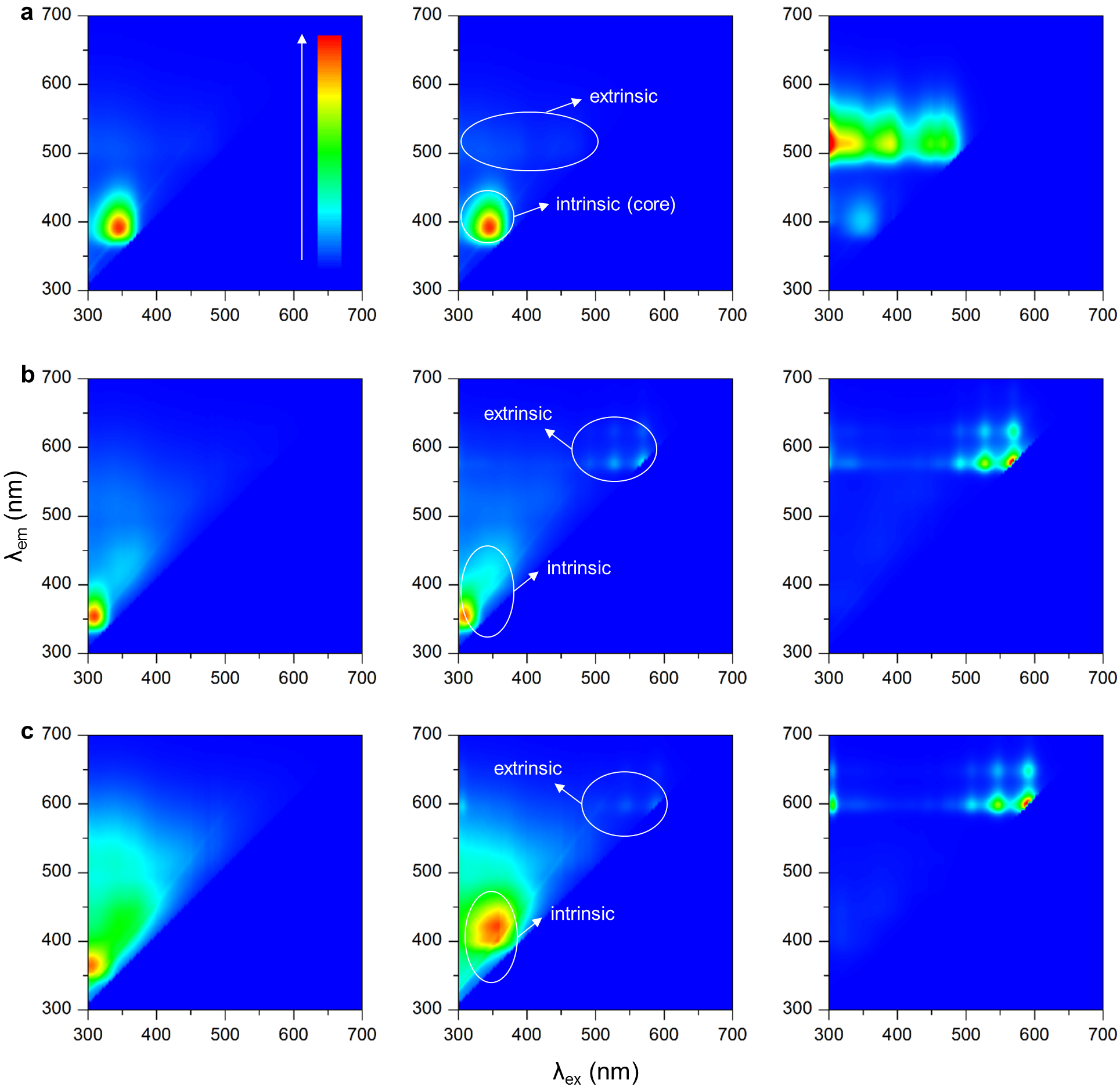


**Figure S9.** Photoluminescence excitation contour maps of **1** (**a**), **2** (**b**) and **3** (**c**) as a function of the degree of functionalization (left column: low; middle column: medium; right column: high).


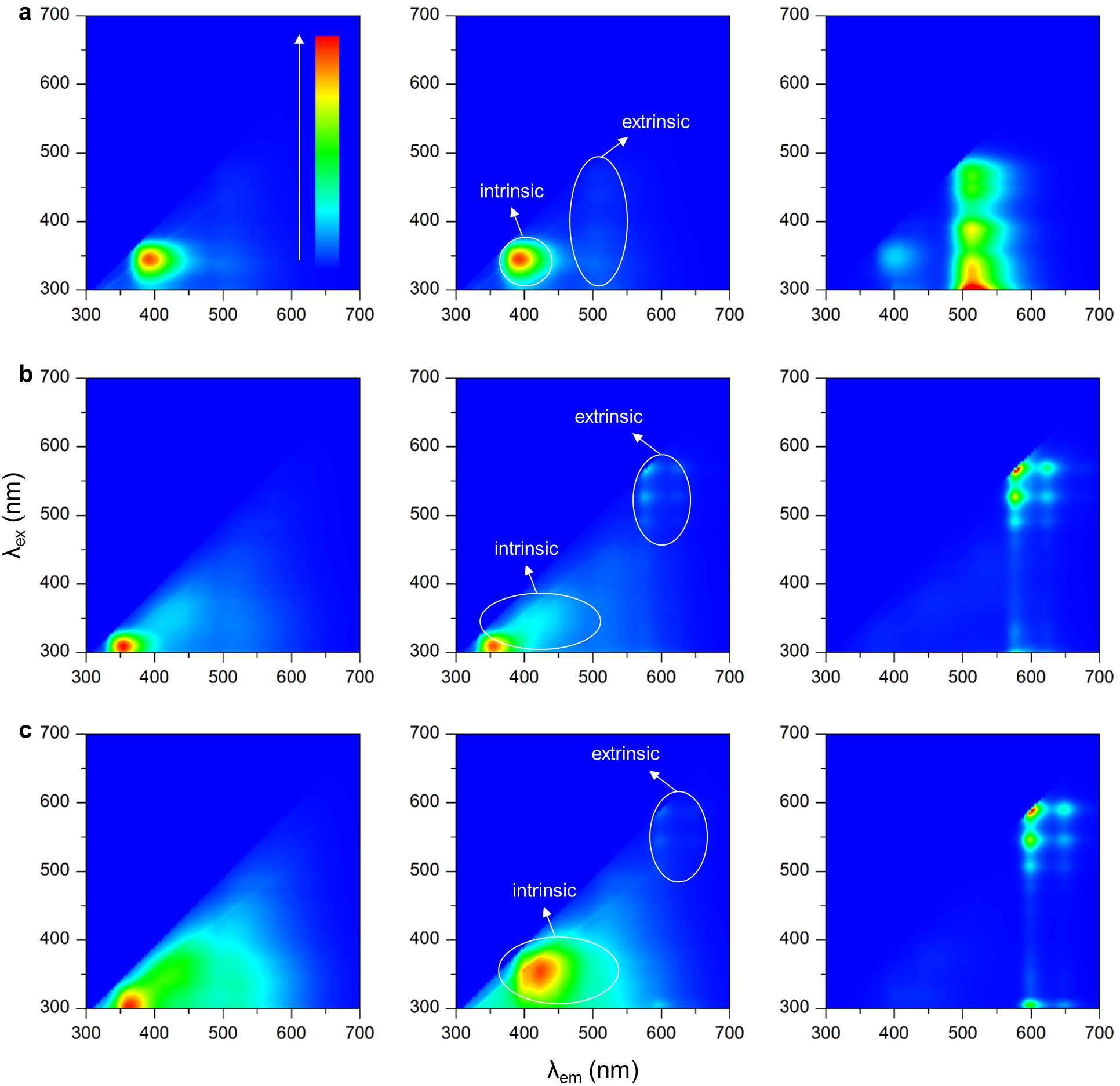


**Figure S10.** Photoluminescence emission contour maps of **1** (**a**), **2** (**b**) and **3** (**c**) as a function of the degree of functionalization (left column: low; middle column: medium; right column: high).


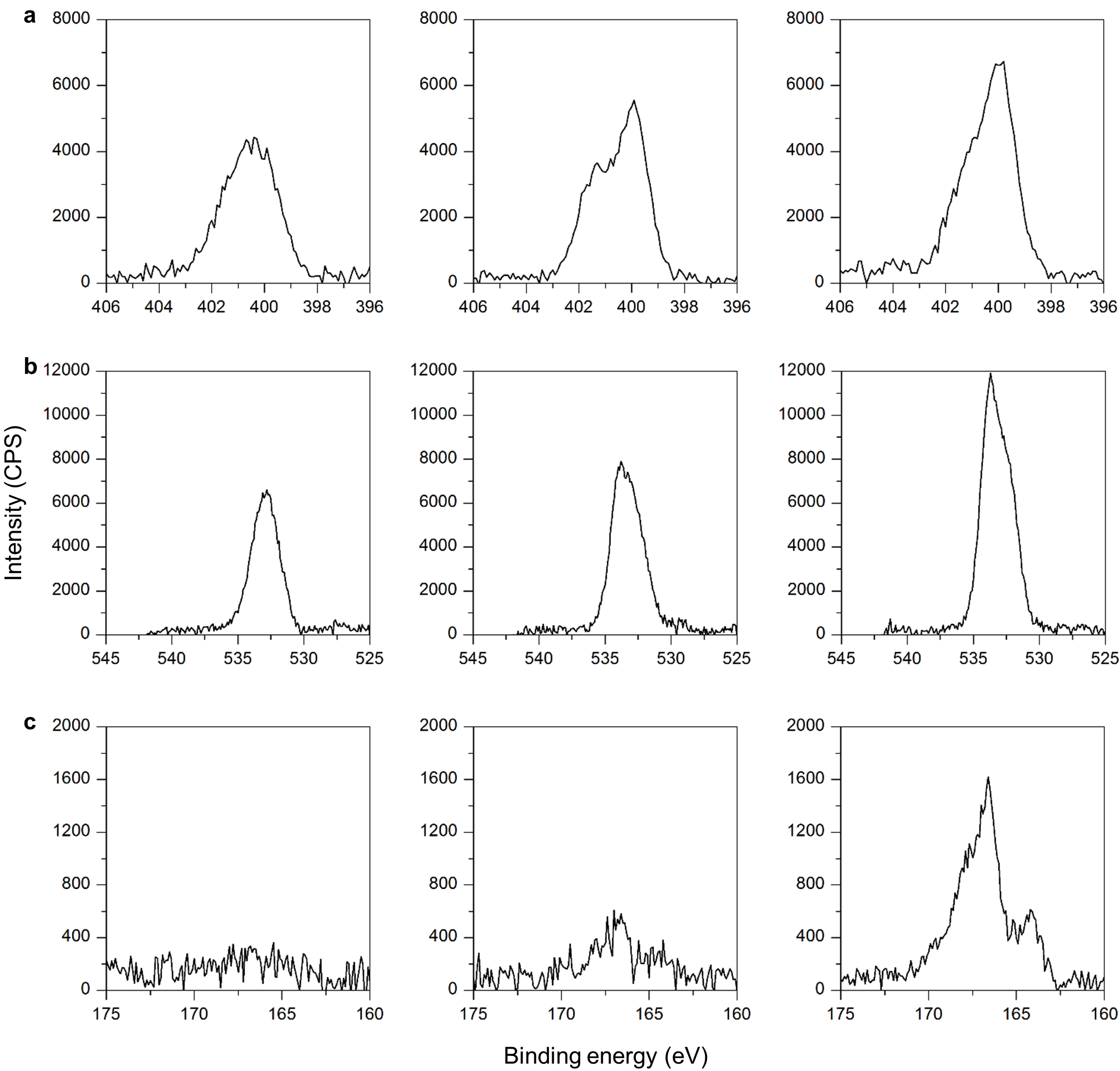


**Figure S11.** X-ray photoelectron spectra of **1** (N1s, **a**), **2** (O1s, **b**) and **3** (S2p, **c**) as a function of the degree of functionalization (left column: low; middle column: medium; right column: high).


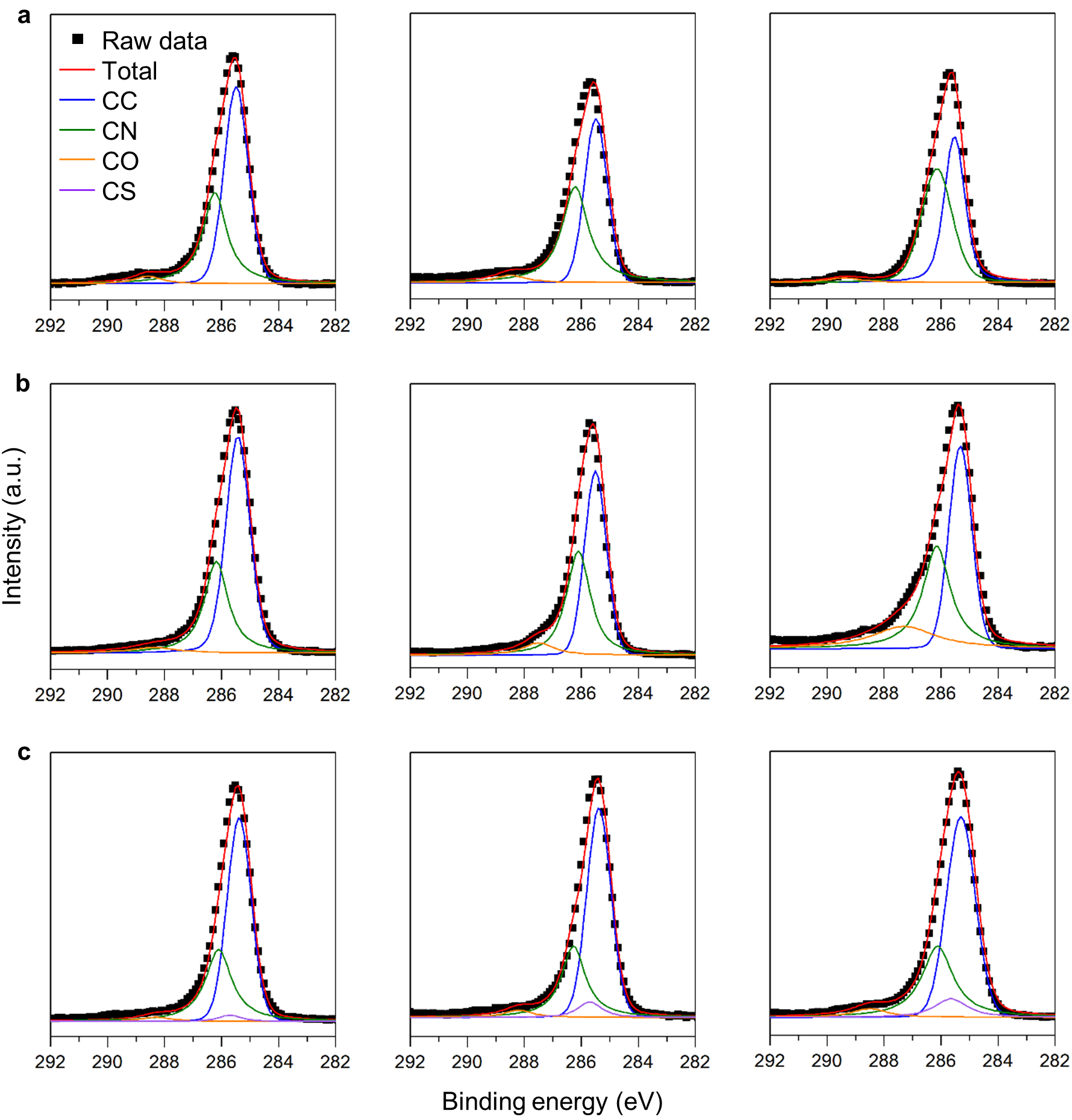


**Figure S12.** X-ray photoelectron spectra (C1s) of **1** (**a**), **2** (**b**) and **3** (**c**) as a function of the degree of functionalization (left column: low; middle column: medium; right column: high).

**Table S2.** Atomic ratios of our functionalized GQDs as a function of the degree of functionalization.

| Sample | Degree | Atomic ratio (%) | | | |
| --- | --- | --- | --- | --- | --- |
|  |  | C1s | N1s | O1s | S2p |
| **1** | Low | 75.10 | 9.76 | 11.49 | ~0 |
|  | Medium | 76.42 | 10.87 | 11.89 | ~0 |
|  | High | 75.75 | 12.47 | 11.45 | ~0 |
| **2** | Low | 81.55 | 5.64 | 11.04 | ~0 |
|  | Medium | 80.39 | 5.88 | 11.99 | ~0 |
|  | High | 79.47 | 6.86 | 12.88 | ~0 |
| **3** | Low | 81.79 | 5.21 | 8.75 | 0.73 |
|  | Medium | 81.86 | 5.58 | 9.94 | 0.99 |
|  | High | 80.95 | 6.02 | 9.01 | 1.37 |

**
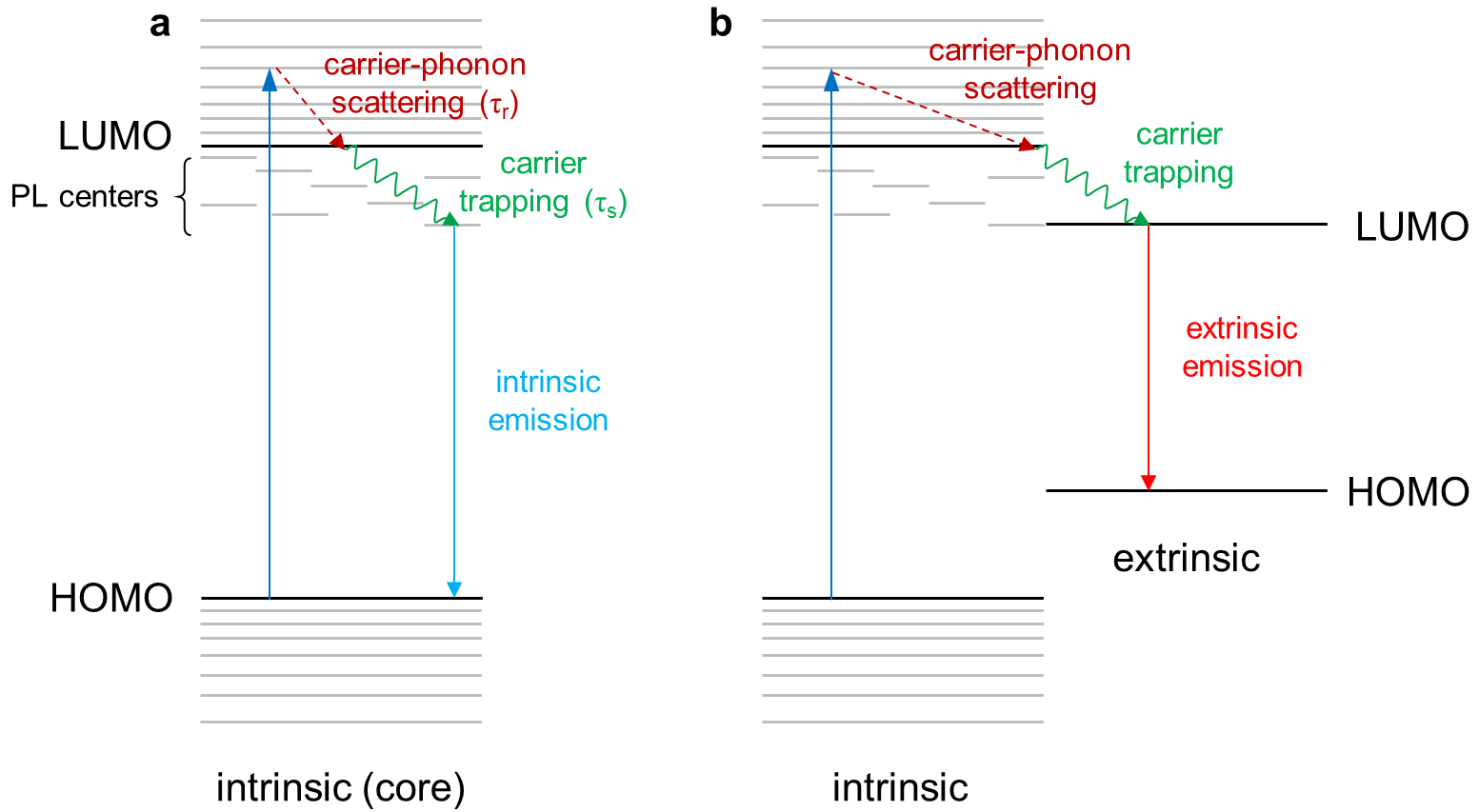
**

**Figure S13.** Proposed electronic structure of bare (**a**) and functionalized (**b**) GQDs.

**Table S3.** Decay times of photoexcited electrons in GQDs.

| Sample | λ_pr_ (nm) | τ_r_ (ps) | τ_s_ (ps) |
| --- | --- | --- | --- |
| Bare | 500 | 17.6 | 740 |
| **1** | 520 | 4.25 | 219 |
| **2** | 580 | 3.91 | 220 |
| **3** | 600 | 1.31 | 103 |

The decay profiles were fitted to bi-exponential functions, which indicates that the decay of photoexcited carriers would take place through two relaxation pathways. Table S3 summarizes the fitting results. In few picoseconds, the carriers were relaxed to the LUMO level of the GQD core. These carriers were then trapped to the photoluminescence (PL) centers in the core (bare GQDs) or in the ligands (functionalized GQDs). The PL centers in the core have many different energy levels so that can disturb the relaxation process, which results in the long carrier trapping time of bare GQDs. The extrinsic PL centers of the functionalized GQDs were generated by the ligand molecules that have well-defined energy structures. Thus, the functionalized GQDs exhibited relatively shorter carrier trapping times.


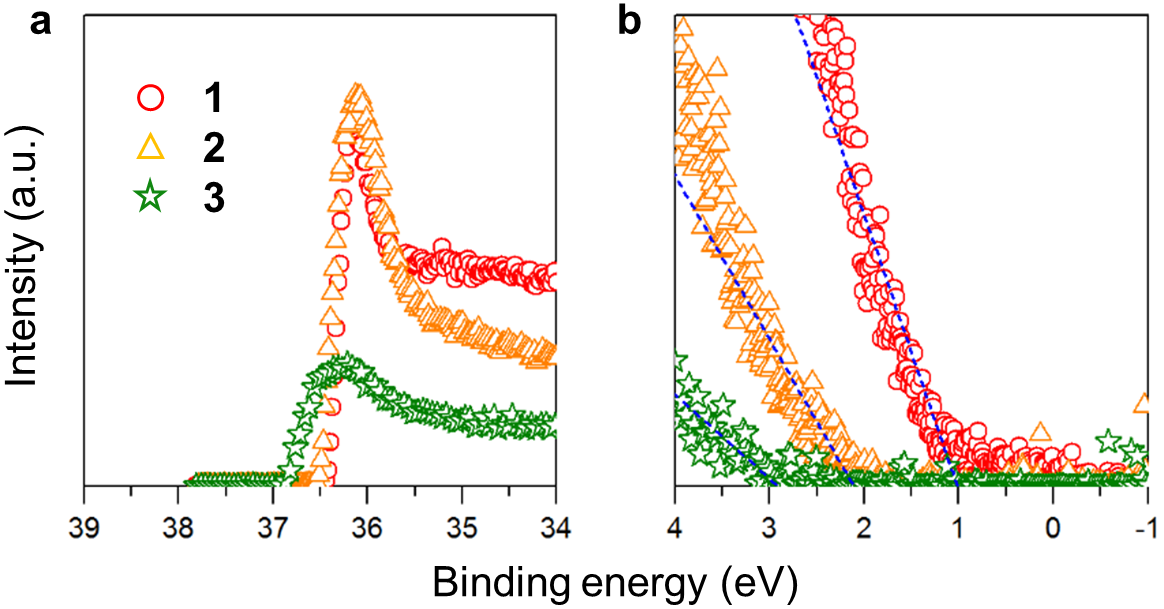


**Figure S14.** Ultraviolet photoelectron spectroscopy data of functionalized GQDs.

Seki and coworkers have suggested a mathematical method to extract the highest occupied molecular orbital (HOMO) level of organic semiconductor deposited on a metal film (typically Au) from ultraviolet photoelectron spectroscopy (S1). The HOMO level (*E*_HOMO_) can be calculated by using the following equation:

*E*_HOMO_ = *ε*_F_ + Φ_m_ – Δ

, where *ε*_F_ is the difference between the Fermi edges of metal and organic semiconductor, Φ_m_ is the work function of metal (5.1 eV for Au), and Δ is the vacuum shift (the difference between the secondary cutoffs of metal and organic semiconductor). We note that *ε*_F_ and Δ are due to higher electron-binding energy of organic semiconductor relative to metal and the presence of a dipole moment at the interface, respectively.


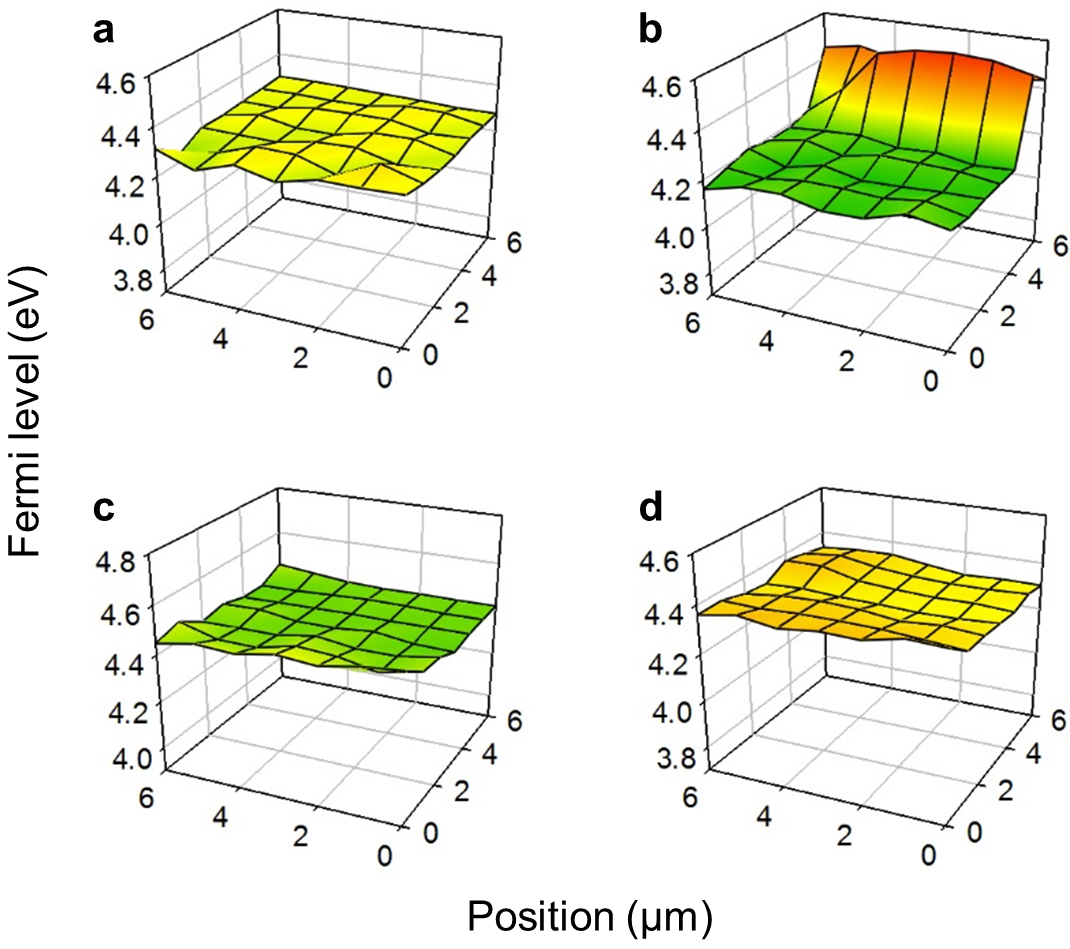


**Figure S15.** Kelvin probe analyses of bare GQDs (**a**), **1** (**b**), **2** (**c**) and **3** (**d**).

The Fermi levels of our GQDs were similar to each other because of their high dipole moments. This result indicates that the HOMO levels of our GQDs were located deeper than 4.2−4.5 eV with respect to the vacuum level.

**Table S4.** Estimation of energy levels of functionalized GQDs.

| Sample | λ_ex,onset_  (nm) | Energy gap  (eV) | HOMO  (eV) | LUMO  (eV) |
| --- | --- | --- | --- | --- |
| **1** | 500 | 2.48 | 5.21 | 2.73 |
| **2** | 560 | 2.21 | 6.60 | 4.39 |
| **3** | 590 | 2.10 | 7.03 | 4.93 |

Photoluminescence excitation onset wavelengths (λ_ex,onset_) were extracted from Fig. 2. These onset values could be matched with the changes in the light absorption spectra (Fig. 1c). The λ_ex,onset_ values were converted into the energy unit (eV) to obtain the energy gaps. The energy gaps were added to the HOMO levels (Fig. S14) to estimate the LUMO levels.

**Table S5.** Comparison with previous literatures.

| ref. | material | light-emitting  mechanism | emission  color | linewidth  (nm) | maximum  efficiency | maximum  luminance  (cd m^−2^) | EQE  (%) |
| --- | --- | --- | --- | --- | --- | --- | --- |
| S2 | carbon  nanodot | phosphor | white | > 100 | 30 lm W^−1^ | - |  |
| S3 | carbon  nanodot | phosphor | blue  orange  white | > 100 | - | - |  |
| S4 | carbon  nanodot | phosphor | white | > 100 | - | - |  |
| S5 | carbon  nanodot | phosphor | yellow  white | > 100 | - | - |  |
| S6 | carbon  nanodot | phosphor | white | > 100 | - | - |  |
| S7 | carbon nanoring | phosphor | white | > 100 | - | - |  |
| S8 | carbon  nanodot | phosphor | white | > 100 | 100 lm W^−1^ | - |  |
| S9 | carbon  nanodot | phosphor | white | > 100 | 108 lm W^−1^ | - |  |
| S10 | carbon  nanodot | phosphor | white | > 100 | - | - |  |
| S11 | GQD | electro-  luminescence | orange | ~100 | - | - |  |
| S12 | carbon  nanodot | electro-  luminescence | white | > 100 | 0.022 cd A^−1^ | ~20 | 0.083 |
| S13 | GQD | electro-  luminescence | white | ~100 | 0.06 cd A^−1^ | ~10 | 0.1 |
| S14 | carbon  nanodot | electro-  luminescence | blue  cyan  magenta  white | > 100 | 0.018 cd A^−1^ | 61 | - |
| S15 | GQD | electro-  luminescence | blue | ~100 | 0.65 cd A^−1^ | ~1000 | - |
| **our work** | **GQD** | **electro-**  **luminescence** | **green**  **orange**  **red** | **~50** | **3.47 cd A^−1^** | **390** | **1.28** |


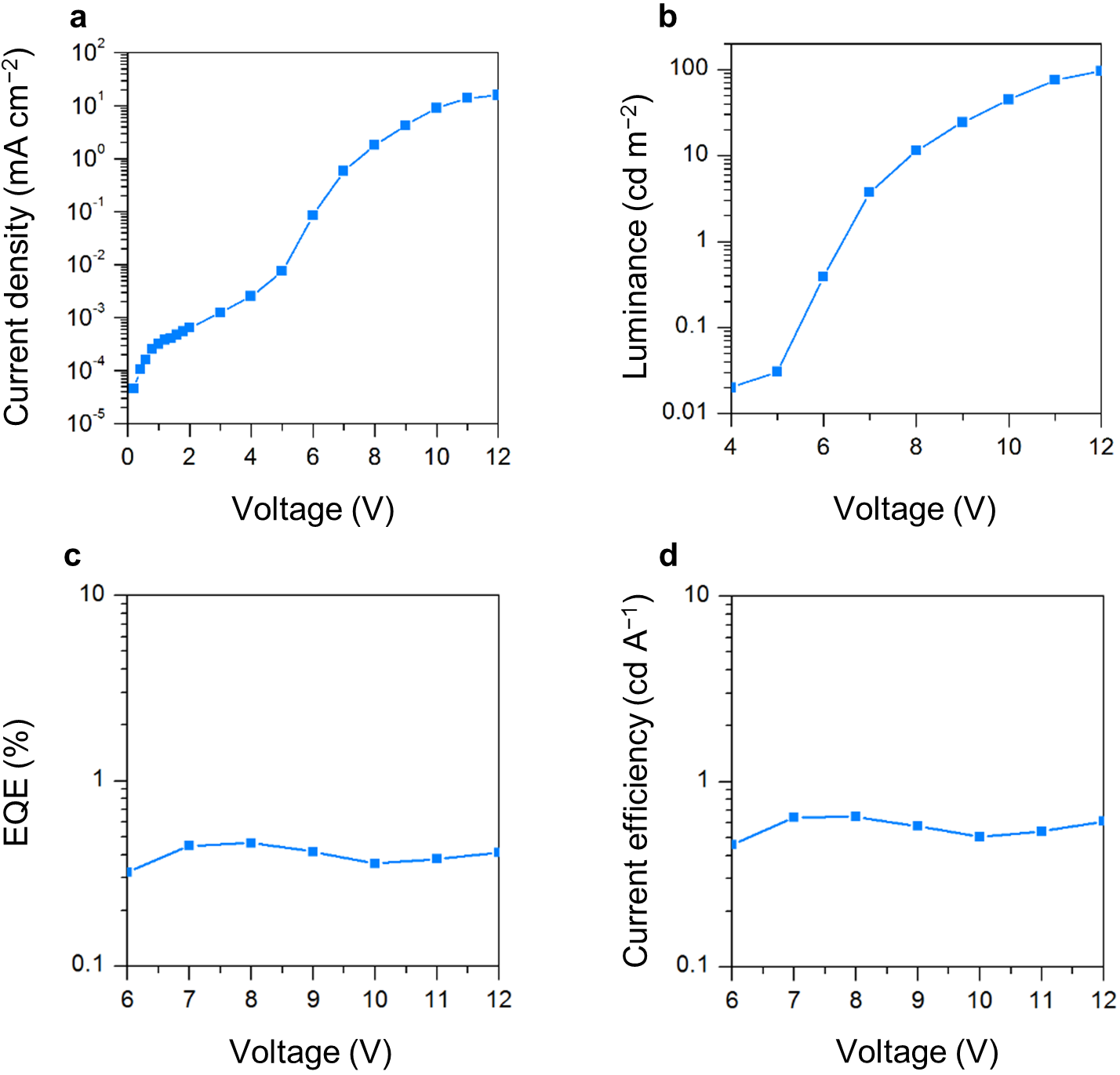


**Figure S16.** Current density versus voltage (**a**), luminance versus voltage (**b**), EQE versus voltage (**c**) and current efficiency versus voltage (**d**) characteristics of host-only LEDs.


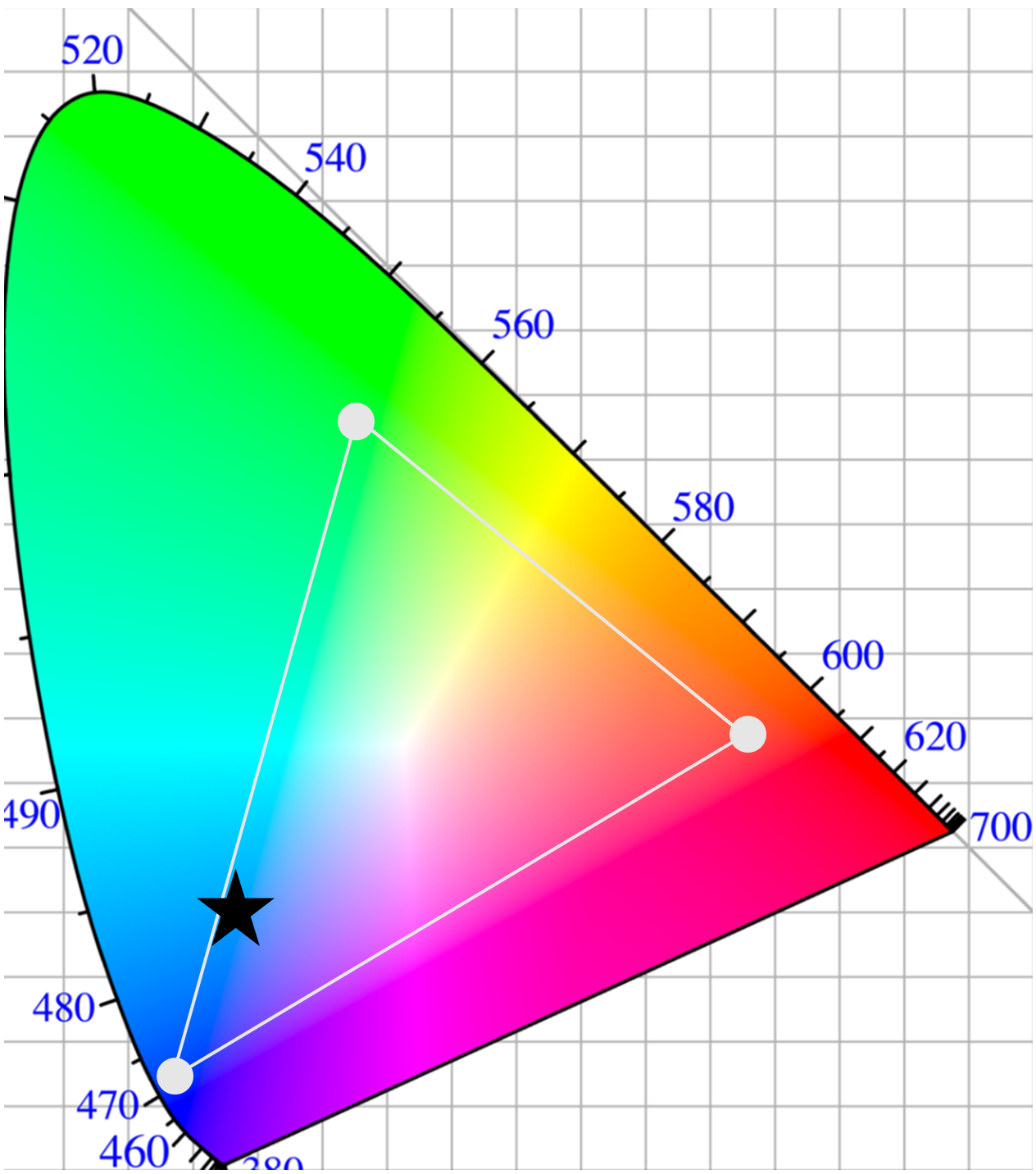


**Figure S17.** CIE coordinates of host-only LEDs. The gray dots and lines represent the National Television System Committee standard color triangle.


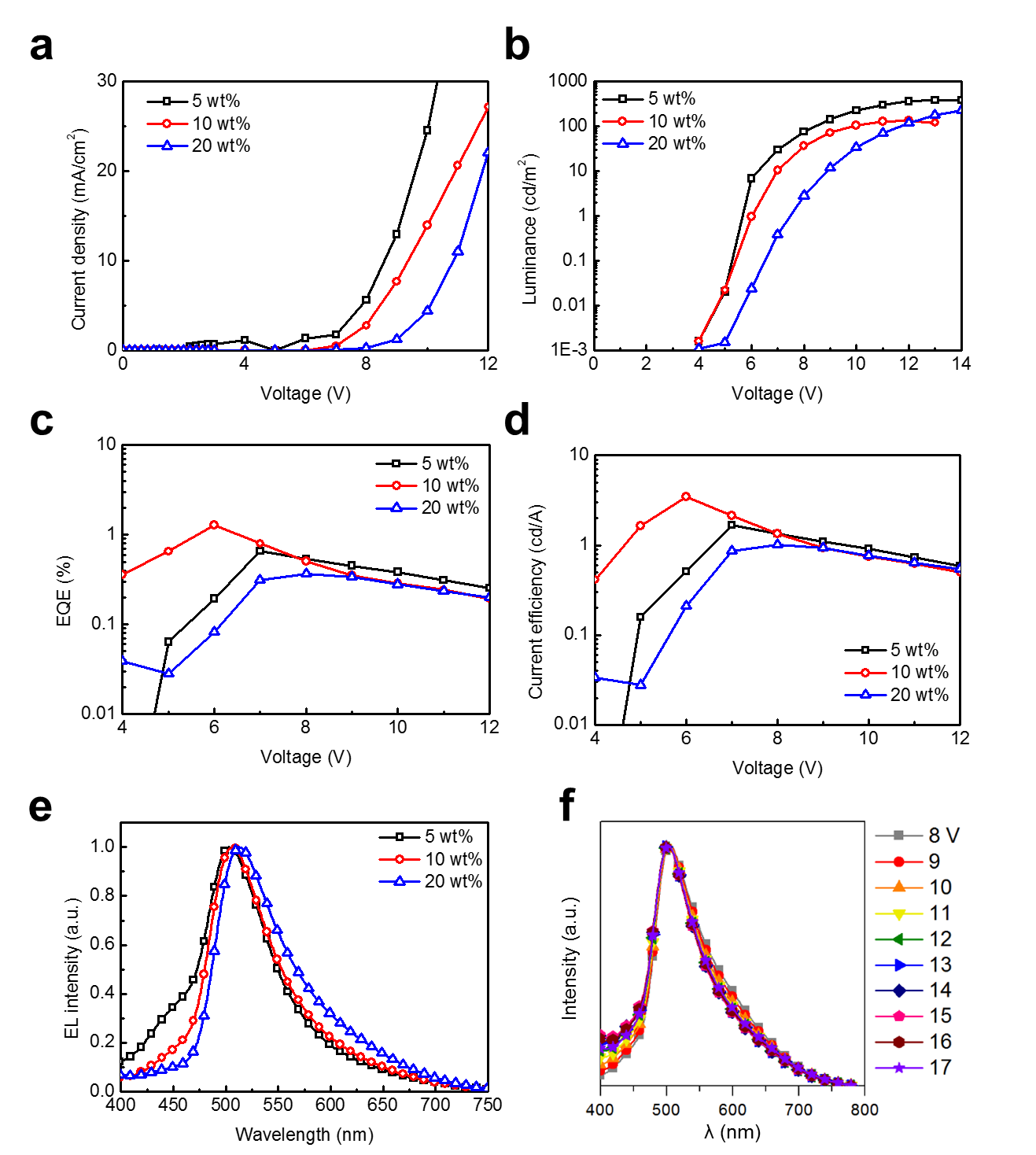


**Figure S18.** Current density versus voltage (**a**), luminance versus voltage (**b**), EQE versus voltage (**c**), current efficiency versus voltage (**d**), and intensity versus wavelength (**e**) characteristics as a function of the concentration of **1** in the host matrix of **1** LEDs. Voltage-dependent electroluminescence spectra of **1** LEDs (**f**).


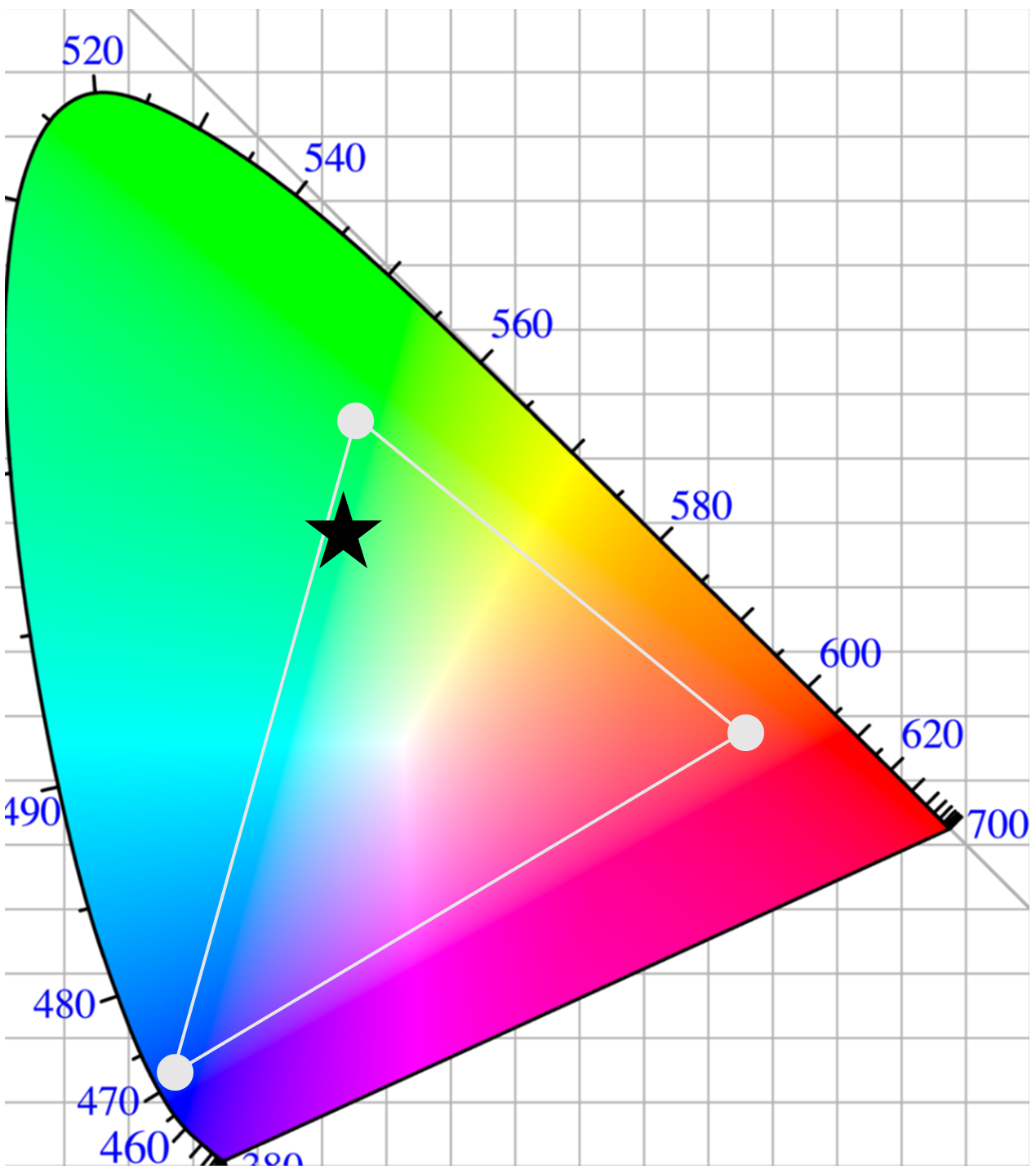


**Figure S19.** CIE coordinates of **1** LEDs. The gray dots and lines represent the National Television System Committee standard color triangle.


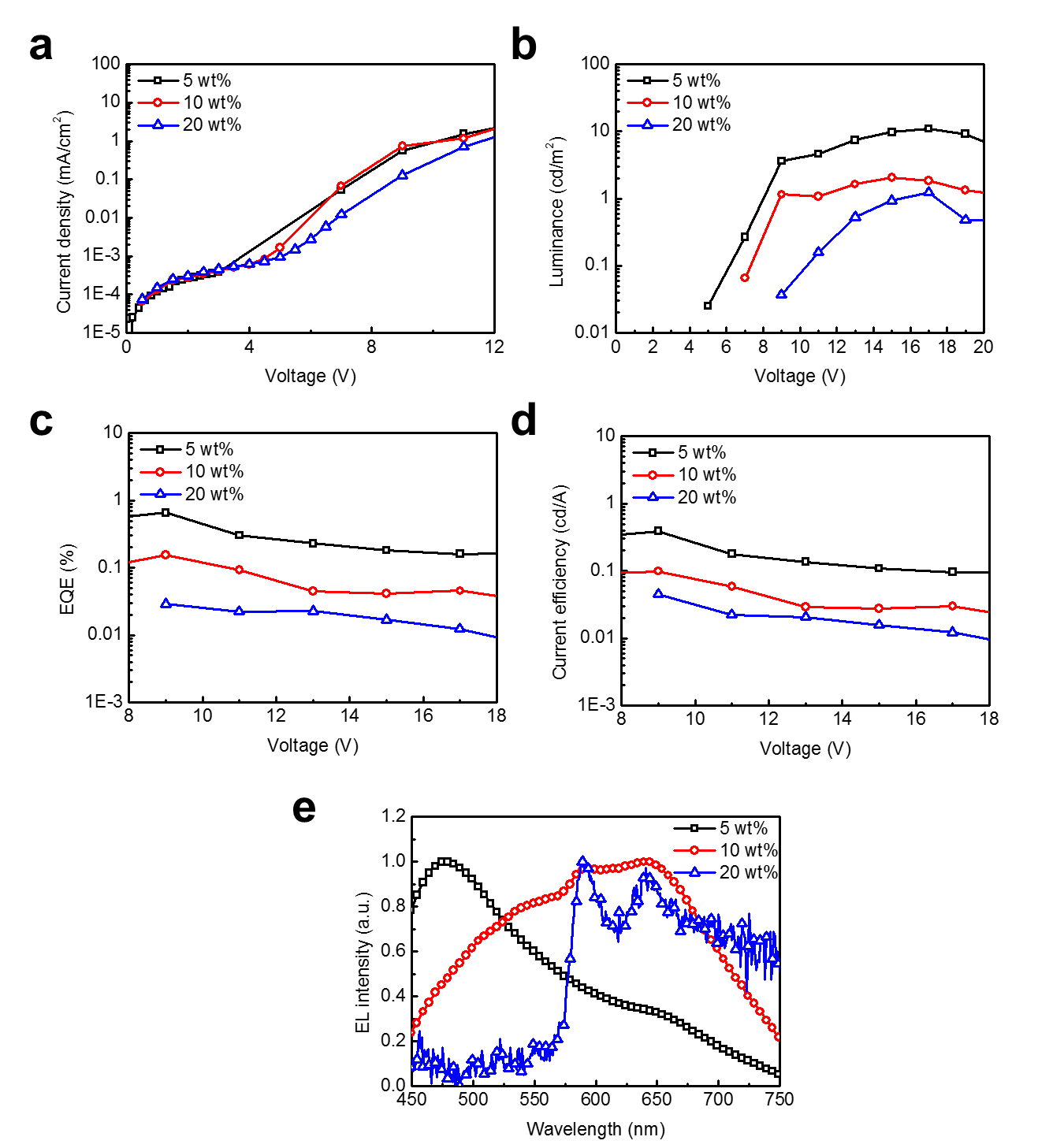


**Figure S20.** Current density versus voltage (**a**), luminance versus voltage (**b**), EQE versus voltage (**c**), current efficiency versus voltage (**d**), and intensity versus wavelength (**e**) characteristics as a function of the concentration of **2** in the host matrix of **2** LEDs.


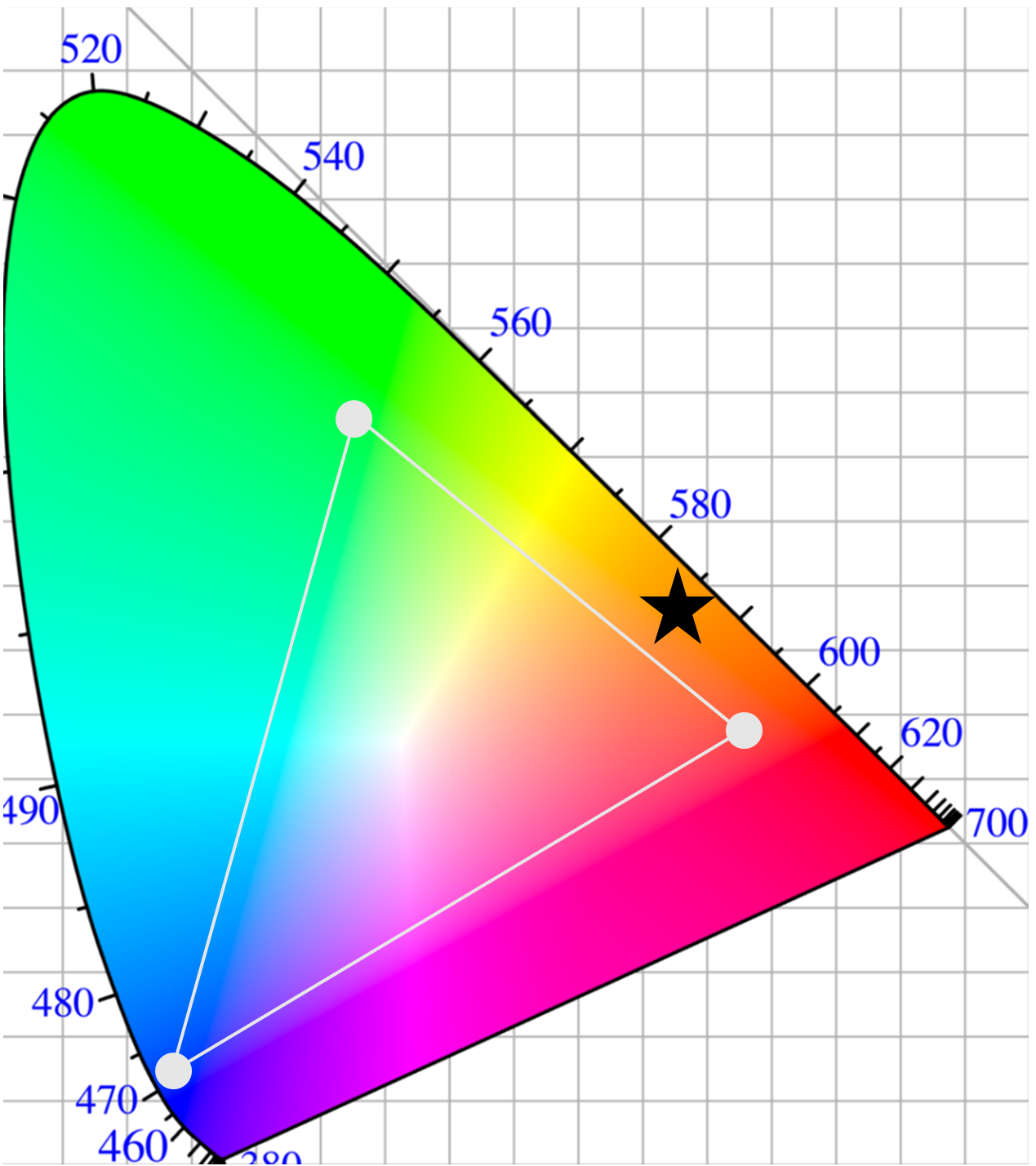


**Figure S21.** CIE coordinates of **2** LEDs. The gray dots and lines represent the National Television System Committee standard color triangle.


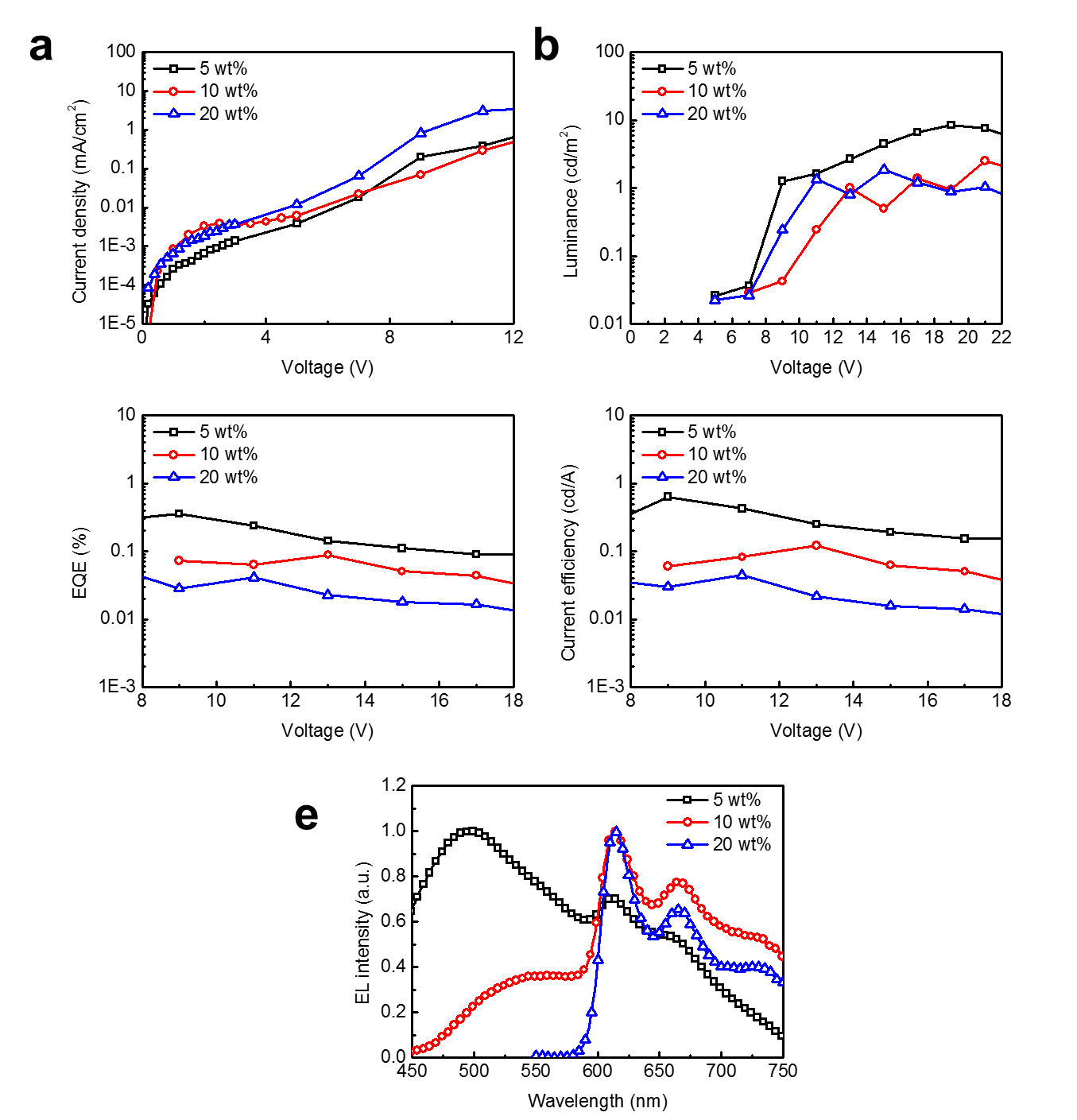


**Figure S22.** Current density versus voltage (**a**), luminance versus voltage (**b**), EQE versus voltage (**c**), current efficiency versus voltage (**d**), and intensity versus wavelength (**e**) characteristics as a function of the concentration of **3** in the host matrix of **3** LEDs.


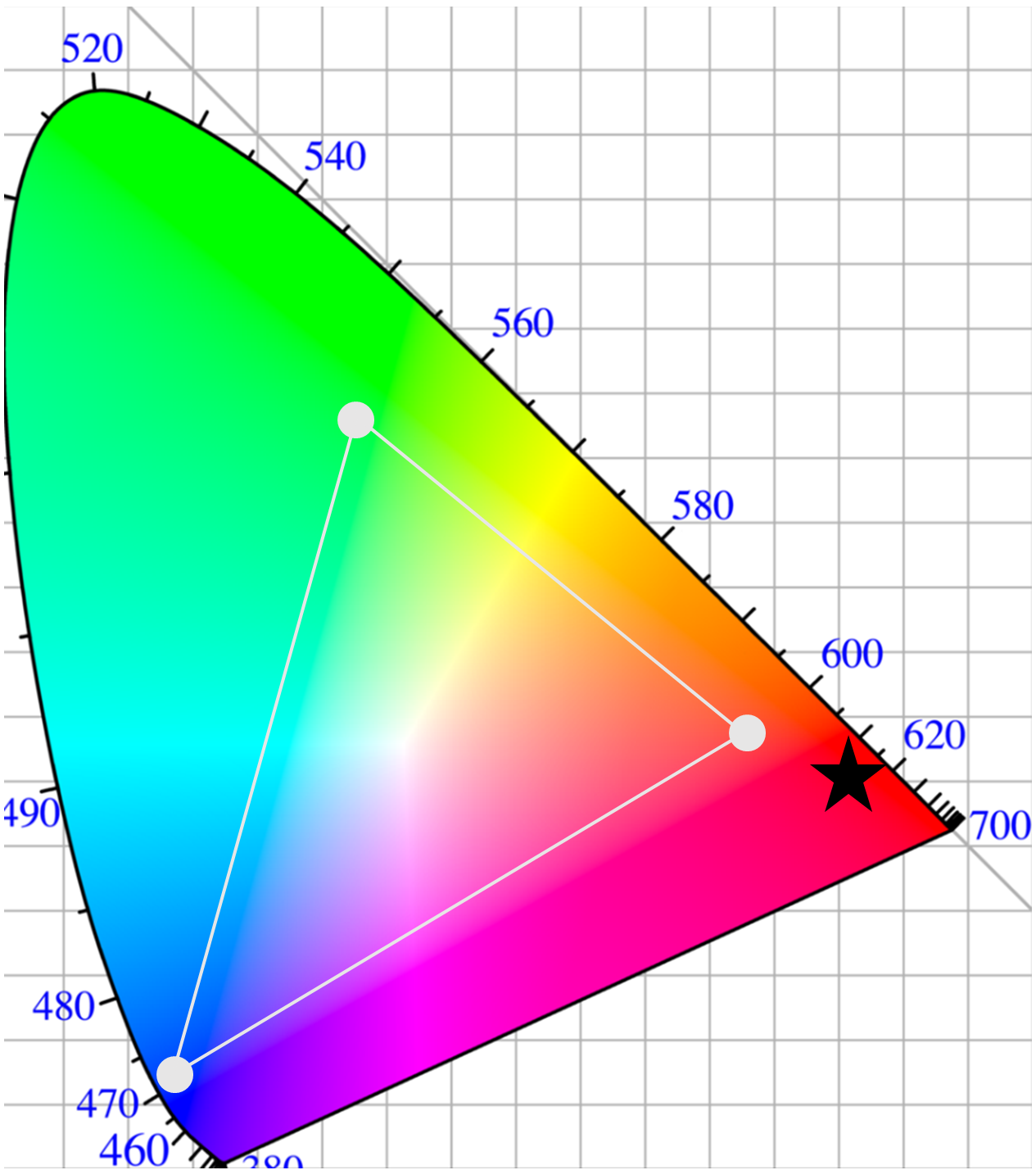


**Figure S23.** CIE coordinates of **3** LEDs. The gray dots and lines represent the National Television System Committee standard color triangle.


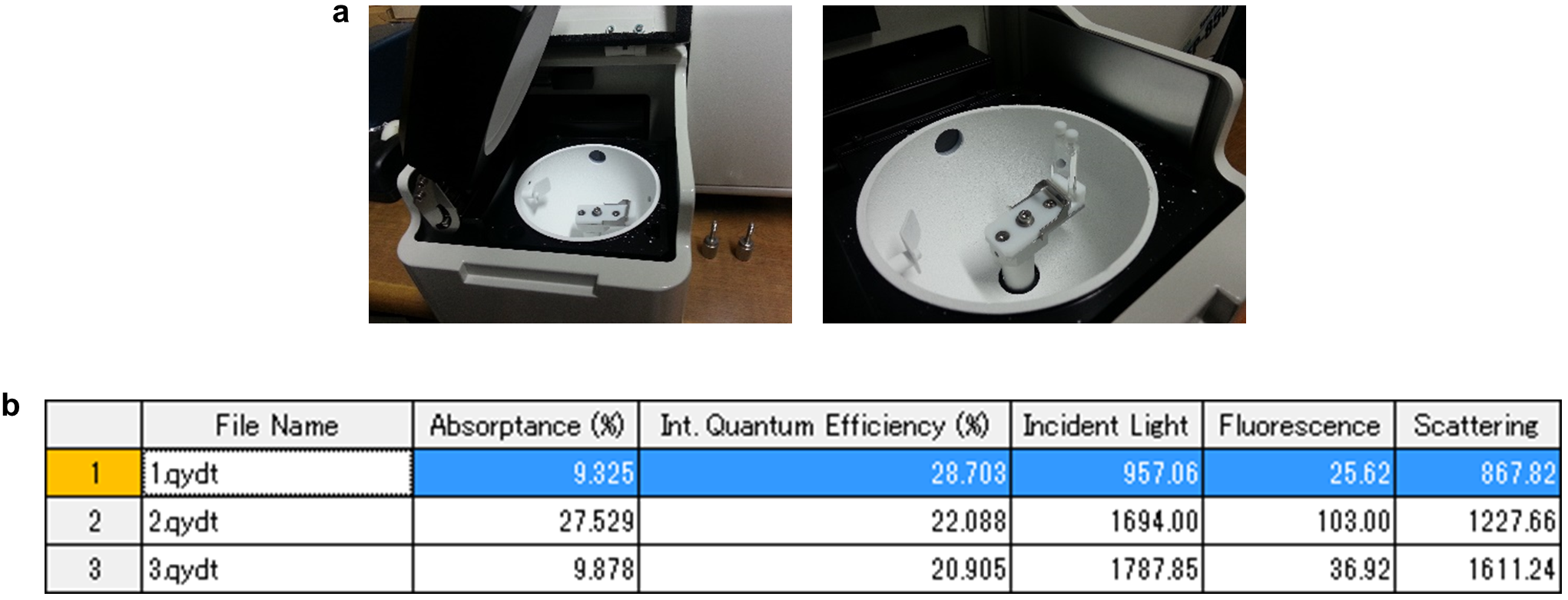


**Figure S24.** (**a**) Photograph of the integrating sphere setup used in the measurements (**b**) Absolute fluorescence quantum yields of functionalized GQDs calculated by using Jasco Spectra Manager II Software.

**References for Supplementary Information**

(S1) Ishii, H. & Seki, K. Energy level alignment at organic/metal interfaces studied by UV photoemission: breakdown of traditional assumption of a common vacuum level at the interface. *IEEE Trans. Electron Devices* **44**, 1295-1301 (1997).

(S2) Li, C.-X., Yu, C., Wang, C.-F. & Chen, S. Facile plasma-induced fabrication of fluorescent carbon dots toward high-performance white LEDs. *J. Mater. Sci.* **48**, 6307-6311 (2013).

(S3) Guo, X., Wang, C.-F., Yu, Z.-Y., Chen, L. & Chen, S. Facile access to versatile fluorescent carbon dots toward light-emitting diodes. *Chem. Commun.* **48**, 2692-2694 (2012).

(S4) Mao, L.-H. *et al.* Facile access to white fluorescent carbon dots toward light-emitting devices. *Ind. Eng. Chem. Res.* **53**, 6417-6425 (2014).

(S5) Zheng, X. *et al.* Highly luminescent carbon nanoparticles as yellow emission conversion phosphors. *Mater. Lett.* **143**, 290-293 (2015).

(S6) Sun, M. *et al*. Towards efficient solid-state photoluminescence based on carbon-nanodots and starch composites. *Nanoscale* **6**, 13076-13081 (2014).

(S7) Li, X. *et al.* Intercrossed carbon nanorings with pure surface states as low-cost and environment-friendly phosphors for white-light-emitting diodes. *Angew. Chem. Int. Ed.* **54**, 1759-1764 (2015).

(S8) Kwon, W., Lee, G., Do, S., Joo, T. & Rhee, S.-W. Size-controlled soft-template synthesis of carbon nanodots toward versatile photoactive materials. *Small* **10**, 506-513 (2014).

(S9) Kwon, W. *et al.* Freestanding luminescent films of nitrogen-rich carbon nanodots toward large-scale phosphor-based white-light-emitting devices. *Chem. Mater.* **25**, 1893-1899 (2013).

(S10) Do, S., Kwon, W. & Rhee, S.-W. Soft-template synthesis of nitrogen-doped carbon nanodots: tunable visible-light photoluminescence and phosphor-based light-emitting diodes. *J. Mater. Chem. C* **2**, 4221-4226 (2014).

(S11) Gupta, V. *et al.* Luminscent graphene quantum dots for organic photovoltaic devices. *J. Am. Chem. Soc.* **133**, 9960-9963 (2011).

(S12) Wang, F., Chen, Y., Liu, C. & Ma, D. White light-emitting devices based on carbon dots’ electroluminescence. *Chem. Commun.* **47**, 3502-3504 (2011).

(S13) Kwon, W. *et al.* Electroluminescence from graphene quantum dots prepared by amidative cutting of tattered graphite. *Nano Lett.* **14**, 1306-1311 (2014).

(S14) Zhang, X. *et al.* Color-switchable electroluminescence of carbon dot light-emitting diodes. *ACS Nano* **7**, 11234-11241 (2013).

(S15) Song, S. H. *et al.* Highly efficient light-emitting diode of graphene quantum dots fabricated from graphite intercalation compounds. *Adv. Optical Mater.* **2**, 1016-1023 (2014).
